# Curcumin-dependent phenotypic transformation of microglia mediates resistance to pseudorabies-induced encephalitis

**DOI:** 10.1186/s13567-023-01149-x

**Published:** 2023-03-14

**Authors:** Luqiu Feng, Guodong Luo, Yuhang Li, Chen Zhang, Yuxuan Liu, Yanqing Liu, Hongyue Chen, Daoling He, Yan Zhu, Ling Gan

**Affiliations:** 1 College of Veterinary Medicine, Southwest University, Chongqing, 402460 China; 2 Chongqing General Station of Animal Husbandry Technology Promotion, Chongqing, 401120 China

**Keywords:** BV2, Curcumin, PRV, phenotype, AMPK, mitochondria, energy metabolism

## Abstract

Pseudorabies virus (PRV) causes viral encephalitis, a devastating disease with high mortality worldwide. Curcumin (CUR) can reduce inflammatory damage by altering the phenotype of microglia; however, whether and how these changes mediate resistance to PRV-induced encephalitis is still unclear. In this study, BV2 cells were infected with/without PRV for 24 h and further treated with/without CUR for 24 h. The results indicated that CUR promoted the polarization of PRV-infected BV2 cells from the M1 phenotype to the M2 phenotype and reversed PRV-induced mitochondrial dysfunction. Furthermore, M1 BV2 cell secretions induced signalling pathways leading to apoptosis in PC-12 neuronal cells, and this effect was abrogated by the secretions of M2 BV2 cells. RNA sequencing and bioinformatics analysis predicted that this phenotypic shift may be due to changes in energy metabolism. Furthermore, Western blot analysis showed that CUR inhibited the increase in AMP-activated protein kinase (AMPK) phosphorylation, glycolysis, and triacylglycerol synthesis and the reduction in oxidative phosphorylation induced by PRV infection. Moreover, the ATP levels in M2 BV2 cells were higher than those in M1 cells. Furthermore, CUR prevented the increase in mortality, elevated body temperature, slowed growth, nervous system excitation, brain tissue congestion, vascular cuffing, and other symptoms of PRV-induced encephalitis in vivo. Thus, this study demonstrated that CUR protected against PRV-induced viral encephalitis by switching the phenotype of BV2 cells, thereby protecting neurons from inflammatory injury, and this effect was mediated by improving mitochondrial function and the AMPK/NF-κB p65-energy metabolism-related pathway.

**Supplementary Information:**

The online version contains supplementary material available at 10.1186/s13567-023-01149-x.

## Introduction

Pseudorabies virus (PRV) is a neurotropic virus similar to herpes simplex virus type I [1] that typically infects domestic and wild animals. However, it has recently been reported that PRV infection in humans manifests as respiratory dysfunction and acute encephalitis [2–4], posing a huge challenge to public health.

PRV infection of the respiratory tract is followed by viral replication in peripheral tissues, such as the muscle mucosa, which causes infection, invasion of peripheral nerves, and transmission to the central nervous system (CNS) through synapses, leading to viral encephalitis and death [4–6].

Microglia are resident mononuclear macrophages in the CNS and have long pseudopodia [7, 8]. These cells typically play immunological roles in response to infectious pathogens in the CNS and continuously scan the CNS and sense changes in the microenvironment through the sensome. Furthermore, these cells respond rapidly to pathological triggers, such as the invasion of pathogenic microorganisms, neuronal death, and protein aggregation, and eliminate factors through phagocytosis and degradation [9–11]. After detecting pathogenic microorganisms in the CNS, microglia first polarize to the classically activated proinflammatory M1 phenotype, and these cells express CD16, CD32, CD40, and major histocompatibility complex (MHC) class II markers and secrete tumour necrosis factor-α (TNF-α), interleukin (IL)-6, nitric oxide (NO), and reactive oxygen species (ROS) to kill the pathogenic microorganisms [11–13]. After clearing the pathogenic microorganisms, microglia polarize from the classically activated M1 phenotype to the alternately activated M2 phenotype, and these cells express arginase-1 (ARG-1) and mannose receptor (CD206) markers and secrete IL-4, IL-10, and transforming growth factor-β (TGF-β) to promote inflammation resolution [14]. This enables the phenotypic inactivation of proinflammatory cells and plays a role in the repair and maintenance of the CNS to re-establish homeostasis [13, 15, 16]. Acute microglial activation is widely believed to be beneficial under neuroinflammatory conditions by promoting the clearance of neurotoxic agents and restoring tissue homeostasis [17]. However, if inflammation cannot be dissipated in time, the surrounding tissues or cells such as neurons will be lost, and the damaged tissue will develop into an internal stimulus, further exacerbating inflammatory injury [18]. Therefore, it is particularly important to switch from the M1 to M2 phenotype in a timely manner.

Curcumin (CUR), which is a natural compound isolated from turmeric, is excellent for treating neuroinflammation and neurological diseases because of its anti-inflammatory properties and ability to cross the blood‒brain barrier [19, 20]. CUR can interact with multiple molecular targets in microglia, thereby promoting phenotypic shifts and exerting anti-inflammatory effects. These molecular targets include nuclear factor kappa-light-chain-enhancer of activated B cells (NF-κB) [20, 21], Toll-like receptor-4 (TLR-4) [20, 22], haem oxygenase-1 (HO-1) [23], myeloid differentiation primary response 88 (MyD88) [20], phosphoinositide 3-kinase/protein kinase B (PI3K/Akt) [24], and p38 mitogen-activated protein kinase (p38 MAPK) [25].

The phenotypic and functional changes in macrophages are accompanied by dramatic changes in energy metabolism pathways in mitochondria [26, 27]. AMP-activated protein kinase (AMPK) is a key regulator of energy metabolism during inflammation [28]. Proinflammatory M1 microglia mainly rely on aerobic glycolysis and fatty acid synthesis (FAS) to produce adenosine triphosphate (ATP), while anti-inflammatory M2 microglia rely on oxidative phosphorylation (OXPHOS) and fatty acid oxidation (FAO) [26, 29]. In this study, we aimed to assess the role of CUR in mediating resistance to PRV-induced encephalitis. Furthermore, we investigated the contribution of different components, such as mitochondrial function- and AMPK/NF-κB p65-energy metabolism-related pathways, in driving microglial phenotypic transitions.

## Materials and methods

### Cell culture, experimental design, and drug administration

BV2 cells (Rio de Janeiro Cell Bank, Portugal), PC-12 cells, and PK-15 cells (Chinese Academy of Sciences Cell Bank, China) were cultured in Dulbecco’s modified Eagle medium (DMEM; 11,995,065, Gibco, USA) supplemented with 10% foetal bovine serum (FBS; 10099141C, Gibco, USA) and 1% penicillin/streptomycin (15,140,122, Gibco, USA). The cells were grown in a humidified incubator at 37 °C with 5% CO_2_. BV2 cells were infected with PRV for 24 h, followed by CUR (20 μM) (A600346, Sangon Biotech, China), Compound C (AMPK inhibitor, 2.5 μM; HY-13418A, MCE Med Chem Express, USA), or small interfering RNA (siRNA) treatment separately or in combination in serum-free medium for 24 h. The supernatant of the BV2 cells treated with/without PRV and/or CUR was collected and further used to treat PC-12 cells for 24 h. The morphology of BV2 and PC-12 cells was observed using a light microscope (Olympus, Japan).

### Model organisms

Ten healthy BALB/c mice (20–35 g) and 100 Sprague–Dawley rats (6–8 week-old; half male and half female; specific pathogen-free animals; 200 ± 20 g) with no history of PRV infection or immunization were purchased from Chongqing Medical University. All mice and rats were maintained under standard conditions (23 ± 2 °C, 60–70% relative humidity, 12 h light/12 h dark cycle) with ad libitum access to food and water.

### Primary microglial culture

Primary microglia were isolated from postnatal Day 1 BALB/C mice. Briefly, the whole brains of BALB/C mice were minced with ophthalmic scissors and filtered using a 200-mesh pore screen under sterile conditions. The mixed cells were seeded in T25 cell flasks in DMEM/F12 (C11330500BT, Gibco, USA) supplemented with 10% FBS (10099141C, Gibco, USA) and 1% penicillin/streptomycin (15,140,122, Gibco, USA). On Day 14, the mixed cells were detached with 0.25% trypsin (25,200,072, Gibco, USA). The cell suspension was collected in centrifuge tubes and centrifuged at 1500 × *g* (25 ℃) for 8 min. The concentration of the cell suspension was adjusted to 1.0 × 10^6^/mL with cell culture medium for subsequent analysis.

### Viral infection and titration

The PRV strain was purchased from the China Veterinary Microorganism Collection and Management Center (preservation number: CVCCAV25) and propagated as previously described [30]. Briefly, PK-15 cells were grown to 90% confluence and infected with PRV at various multiplicities of infections. After 2 h, the inoculum was removed by aspiration, and the cells were washed twice with PBS. After 3 days, the cytopathic effects on PK-15 cells were observed using an inverted microscope (Olympus, Japan). The median tissue culture infectious dose (TCID_50_) was calculated using the Reed–Muench method [31].

### Transient transfection with siRNA

Several siRNA fragments for AMPKα1 were designed, and those that efficiently inhibited its translation were selected (sense: 5'-GCCGACCCAAUGAUAUCAUTT-3' and antisense: 5'-AUGAUAUCAUUGGGUCGGCTT-3'; Gene Pharma, China). The designed siRNAs were all modified with 2'-O-methyl and 5'-carboxyfluorescein fluorescent labels to avoid degradation and assess transfection efficiency, respectively. The inhibitory efficiency of the siRNA probes was assessed by Western blot analysis of AMPKα1 protein levels. BV2 cells were cultured for 24 h and grown to approximately 70% confluence. Subsequently, the cells were transfected with scrambled siRNA or AMPKα1 siRNA using GP-transfect-Mate (G04008, Gene Pharma, China) according to the manufacturer’s protocol. All assays were performed 24 h after the transfection of siRNA.

### Cell viability, lactic dehydrogenase (LDH) activity and malondialdehyde (MDA) level assessment

BV2 and PC-12 cell viability was assessed using CCK-8 reagent (BS350B, Biosharp, China). BV2 cells were seeded at a density of 5 × 10^4^ cells/well in 96-well plates, incubated overnight and treated with varying concentrations of CUR or Compound C for 24 h. Alternatively, PC-12 cells were seeded at a density of 5 × 10^4^ cells/well in 96-well plates, incubated overnight and exposed to the conditioned media (CM) of different BV2 cell phenotypes. CM was obtained from the supernatant of BV2 cells treated with/without PRV and/or CUR. After the PC-12 cells were disrupted and homogenized by a high-speed disperser, LDH activity and MDA levels were determined by a colorimetric method using LDH and MDA detection kits, respectively (A003-4–1, A020-2–2, Nanjing Jiancheng Biological Company, China) according to the manufacturer’s protocol.

### NO analysis

BV2 cells were seeded in 6-well plates at a density of 5 × 10^5^ cells/mL and incubated overnight. BV2 cells were infected with different PRV titres for 24 h, or the cells were infected with 1.66 × 10^6^ TCID_50_ PRV for 6, 12, and 24 h or 1.66 × 10^6^ TCID_50_ PRV for 24 h, followed by CUR treatment for 6, 12, and 24 h. The supernatant was collected, and NO levels were estimated using an NO detection kit (A003-4–1, A020-2–2, Nanjing Jiancheng Biological Company, China) according to the manufacturer’s protocol.

### Intracellular reactive oxygen species analysis

BV2 cells were seeded in 6-well plates at a density of 5 × 10^5^ cells/mL, incubated overnight and infected with different PRV titres for 24 h, or the cells were infected with 1.66 × 10^6^ TCID_50_ PRV for 6, 12, and 24 h or 1.66 × 10^6^ TCID_50_ PRV for 24 h and then treated with CUR for 6, 12, and 24 h. The fluorescent probe 2,7-dichlorofluorescein diacetate (DCFH-DA) (10 μM) (E004-1–1, Nanjing Jiancheng Biological Company, China) was added to the cells and incubated for approximately 1 h in the dark. The cells were then washed once with PBS, and the fluorescence was measured using a microplate reader (Tecan, Switzerland) at an excitation wavelength of 500 nm and an emission wavelength of 525 nm. After the fluorescence was measured, the cells were observed under a fluorescence microscope (Olympus, Japan). Unstained cells were used as blanks to normalize the fluorescence intensity in the different treatment groups.

### Analysis of cytokines

The levels of cytokines released from BV2 cells and primary microglia in culture were quantified using the following commercial enzyme-linked immunosorbent assay (ELISA) kits according to the manufacturer’s instructions: mouse TNF-α uncoated ELISA kit (88–7324-22, Thermo Fisher, USA), mouse IL-6 uncoated ELISA kit (88–7064-22, Thermo Fisher, USA), mouse IL-4 uncoated ELISA kit (88–7044-22, Thermo Fisher, USA), and mouse IL-10 uncoated ELISA kit (88–7105-22, Thermo Fisher, USA).

### Flow cytometry

BV2 cells and primary microglia were collected after the indicated treatments and stained with fluorescence-conjugated monoclonal antibodies according to the manufacturer’s instructions. Cultured and treated cells were resuspended in PBS solution. Then, a 95-μL cell suspension was obtained, and 5 μL of FITC-anti-mouse CD40 (11–0402-81, Thermo Fisher, USA), APC-anti-mouse CD206 (17–2061-82, Thermo Fisher, USA), or CoraLite® 488 anti-mouse CD11b (CL488-65,055, Proteintech, China) was added. The suspension was incubated for 2 h at 4 °C in the dark, fixed with 2% paraformaldehyde for 30 min and permeabilized with 0.1% Triton X-100 for 15 min. Finally, the cells were blocked with 1% bovine serum albumin (BSA, BS114-100g, Biosharp, China) for 1 h and washed three times with PBS buffer. Subsequently, 5 μL of PE-anti-mouse CD16/32 (12–0161-82, Thermo Fisher, USA) or PE-anti-mouse Arg-1 (12–3697-82, Thermo Fisher, USA) and CoraLite® 647 anti-mouse MHC Class II (CL647-65,122, Proteintech, China) were added, and the suspensions were incubated at 4 °C for 2 h in the dark. Then, the cells were washed three times with prechilled 1% BSA solution. Finally, flow cytometry was performed using an Accuri C6 flow cytometer (BD Biosciences, San Jose, CA, USA). The data were analysed using FlowJo™ v10 software (for Windows) Version 10 (Ashland, BD Life Sciences).

### Transmission electron microscopy (TEM)

Mitochondrial morphology was examined using TEM. BV2 cells were added to a 2.5% glutaraldehyde fixative solution and stored overnight at 4 °C. Images were captured using TEM (Hitachi-7500, Japan).

### Mitochondrial membrane potential (MMP) measurement

BV2 cells were cultured overnight in 12-well plates at a density of 2.5 × 10^5^ cells/well. MMP was measured using a JC-1 assay kit (BL726A, Biosharp, China). BV2 cells were incubated with 10 μM JC-1 dye for 20 min at 37 °C (shielded from light) and washed with PBS prior to being evaluated using a microplate reader (Tecan, Switzerland) and fluorescence microscope (Olympus, Japan). In normal mitochondria, JC-1 forms aggregates that emit red fluorescence (561 nm). Following a decrease or loss of MMP, aggregated JC-1 is released into the cytoplasm in its monomeric form, which emits green fluorescence (488 nm).

### Transcriptome sequencing analysis and real-time PCR

RNA-seq analysis was performed by Allwegene (Allwegene, China). RNA (three biological replicates per group) was extracted from BV2 cells using TRIzol reagent (B511311, Sangon Biotech, China) according to the manufacturer’s instructions, and a cDNA library was prepared using a *PerfectStart*® Uni RT**&**qPCR Kit (TransGene Biotech, China) with 1000 ng of total RNA. cDNA, primers, *PerfectStart* Green qPCR SuperMix, and nuclease-free water were combined into a 20 µL reaction system to perform quantitative real-time PCR on a real-time PCR system (QuantStudio 5, Thermo Scientific, USA). RNA-seq was performed using the Illumina HiSeq 2500 platform. A corrected *p* value of 0.05 and a log2 (fold change) of 1 were set as thresholds for significantly differentially expressed genes (DEGs). Gene Ontology (GO) and Kyoto Encyclopaedia of Genes and Genomes (KEGG) pathway enrichment functional analyses were performed on the DEGs in each group by using GOSeq software and KOBAS software [32, 33].

The following primer pairs were designed to measure transcript abundance relative to β-actin (B661302, Sangon Biotech, China) as an internal reference: malonyl-CoA-acyl carrier protein transacylase (*Mcat*): TCTGGTTTCTGTCTACTCCAAC (F) and CCTTTCGTATATGGCATGCATC (R); lactate dehydrogenase A (*Ldha*): AAGACTACTGTGTAACTGCGAA (F) and ACTTGAAGATGTTCACGTTTCG (R); hexokinase 1 (*Hk1*): ATTAAGAAGCGAGGGGACTATG (F) and CTCCCCATTCCGTGTTAATACA (R); phosphofructokinase (*Pfkl*): ACGGTATACATCGTGCATGAT (F) and GATGTTGTAGGTGCGGAGATTC (R); and histocompatibility 2, class II, locus Mb1 (*H2dmb1*): CATGGGCCGAAAATTTTTCAAG (F) and CTCCCTTGTGTTAAAAGGTGTG (R).

### Detection of the oxygen consumption rate (OCR) in BV2 cells

The cellular OCR was measured using an OCR assay kit (600,800; Cayman Chemical, USA). BV2 cells were seeded into 96-well plates and incubated overnight. After treatment, the medium was discarded, and 10 μL of the phosphorescent oxygen probe and 100 μL of LHS mineral oil assay reagent (prewarmed at 37 °C) were added to each well. A microplate reader (Tecan, Switzerland) was used, and the ratiometric time-resolved fluorescence (lifetime) measurement mode at an excitation wavelength of 380 nm and emission wavelength of 650 nm was selected for dynamic measurement for 2 h.

### Detection of the extracellular acidification rate (ECAR) in BV2 cells

The ECAR was measured using a glycolytic cell-based assay kit (600,450; Cayman Chemical, USA). BV2 cells were seeded into 96-well plates and incubated overnight. After the different groups were treated, the medium was collected and centrifuged at 400 × *g* for 5 min at 4 °C. An aliquot of the supernatant from each sample was added to the reaction solution and gently shaken for 30 min at 25 °C on an orbital shaker. The absorbance was measured at 490 nm using a microplate reader (Tecan, Switzerland).

### Determination of ATP levels in BV2 cells

ATP levels were measured using a firefly luciferase-based ATP detection kit (S0026, Beyotime, China). After being washed with PBS, BV2 cells were lysed using ATP detection lysis buffer, followed by centrifugation at 12 000 × *g* for 5 min at 4 °C, and the supernatant was collected. Then, 100 µL of the supernatant was mixed with 100 µL of ATP detection solution in a 1.5 mL tube. Luminescence (corresponding to total ATP levels) was immediately measured in relative light units (RLU) (nmol/mg) using a Turner Biosystems luminometer (Tecan, Switzerland). Finally, the ATP level of each sample was determined according to the RLU value of the standard sample and normalized to the protein concentration.

### Western blotting

BV2 and PC-12 cells were lysed in ice-cold radioimmunoprecipitation assay buffer (RIPA; BL504A, Biosharp, China), 1 × complete protease inhibitor (BL612A, Biosharp, China), and a phosphatase inhibitor cocktail (BL615A, Biosharp, China). Cell homogenates (20 μg/well) were loaded onto 8%, 10%, or 12% SDS polyacrylamide gels under denaturing conditions. Proteins were resolved electrophoretically at 100 mA for 90 min and transferred onto a 0.45 μm polyvinylidene fluoride (PVDF) membrane (Millipore, USA) at 200 V for 60 min (Power Pack; Bio-Rad Laboratories, USA). Furthermore, the membrane was blocked with 5% nonfat dry milk or 5% BSA in tris-buffered saline containing Tween-20 (TBST) at 25 ℃ and then incubated overnight at 4 °C with the following antibodies: rabbit anti-AMPK (1:2000, bs-1115R, Bioss, China), phosphor(p)-AMPK^Thr172^ (1:1000, 2531, Cell Signaling, USA), lactate dehydrogenase A (LDHa, 1:1000, WL03271, Wanleibio, China), nuclear factor kappa-B p65 (NF-κB p65, 1:1000, WL01273b, Wanleibio, China), p-NF-κB p65^Ser536^ (1:10 000, WL02169, Wanleibio, China), glycerol-3-phosphate acyltransferase 4 (GPAT4, 1:1000, bs-15587R, Bioss, China), Bax (1:10 000, 50,599–2-Ig, Proteintech, China), Caspase-3/p17/p19 (1:1000, 19,677–1-AP, Proteintech, China), Bcl-2 (1:4000, 26,593–1-AP, Proteintech, China), and β-actin (1:5000, 20,536–1-AP, Proteintech, China). Then, the membranes were washed with TBST, clipped according to the prestained protein ladder (BL712A, Biosharp, China), and incubated with horseradish peroxidase-conjugated goat anti-rabbit IgG (H + L) (1:10 000, SA00001-2, Proteintech, China). An enhanced chemiluminescence kit (P0018AS, Beyotime, China) was used to visualize the immunoblots. Immunoreactive bands were analysed using ImageJ software [34]. β-actin was used as the loading control.

### Apoptosis assay

PC-12 cells were exposed to the CM of BV2 cells with different phenotypes for 24 h. The cells were then washed with PBS, centrifuged, and resuspended in 200 μL of binding buffer. Then, 5 μL of Annexin V-FITC (BL107A, Biosharp, China) was added, mixed gently and incubated at 25 ℃. After 15 min, 10 μL of propidium iodide staining solution was added to the cells, mixed, covered with aluminium foil and incubated for 10–20 min at 25 ℃ (in the dark). Cells were then observed under a fluorescence microscope (Olympus, Japan) for 1 h.

### Groupings and drug administration in rats

Forty Sprague–Dawley rats were randomly divided into four groups, with 10 rats in each group (half male and half female). Rats in the PRV-infected groups were intraperitoneally injected with three different titres of TCID_50_ PRV solution (2.85 × 10^2^, 2.85 × 10^3^, and 2.85 × 10^4^), and each rat was injected with a volume of 0.1 mL. The control group was intraperitoneally injected with the same volume of DMEM. When rats in the PRV group had been infected for 7 days, the infection dose with a survival rate higher than 60% was selected as the follow-up test dose [5].

Sixty Sprague–Dawley rats were randomly divided into six groups with 10 rats in each group (half male and half female), which included the control, PRV, RES, CUR L, CUR M, and CUR H groups. The RES group was injected with 50 mg/kg bw resveratrol, the CUR L group was injected with 25 mg/kg bw curcumin solution, the CUR M group was injected with 50 mg/kg bw curcumin solution, the CUR H group was injected with 100 mg/kg bw curcumin solution, and the control and PRV groups were intraperitoneally injected with the same amount of 0.5% sodium carboxymethyl cellulose solution once per day for a total of 14 days. On the 8^th^ day, each rat in the PRV, RES, CUR L, CUR M, and CUR H groups was intraperitoneally injected with 0.1 mL of 2.85 × 10^3^ TCID_50_ PRV solution.

### Open-field test

Open-field test experiments were performed on the. A transparent glass box (60 × 60 × 40 cm^3^) was divided into 36 equal squares with a 0.5 cm-wide medical bandage. Then, the rats were placed in the transparent glass box, and a video imager (Xiaomi, China) was used to record the number of grids that the rats in each group moved horizontally within for 5 min (effective movement was considered when more than three legs were in a square) and the number of times the rats stood (taking the forelimb off the ground represented effective standing).

### Measurement of body temperature, body weight, and the viscera index

From 1 to 7 days post-infection (dpi), rat body weight and temperature were measured. The organ index was evaluated by measuring the ratio of the weight of the brain tissue to the body weight of the rat at 7 dpi.

### Preparation of pathological sections

At 7 dpi, the cerebral cortices of the rats were collected, sectioned into 5 × 5 × 3 mm sections and processed in tissue cassettes according to a standard protocol. Glass slides were prepared for microscopic examinations using 5-μm sections of formalin-fixed paraffin-embedded (FFPE) tissues with routine haematoxylin and eosin (HE) staining. The sections of each brain region were observed under an inverted microscope (Olympus, Japan).

### Statistical analysis

Statistical analyses were performed using SPSS 23.0 statistical software (IBM, Armonk, NY, USA). Quantitative data were examined using one-way analysis of variance (ANOVA) followed by a least significant difference (LSD) post hoc test for multiple comparisons among the groups, and a two-tailed independent t test was used to compare the differences between two groups. Differences were considered statistically significant at *P* < 0.05.

## Results

### PRV-infected BV2 cells exhibit increased levels of inflammatory markers

**Figure 1:**
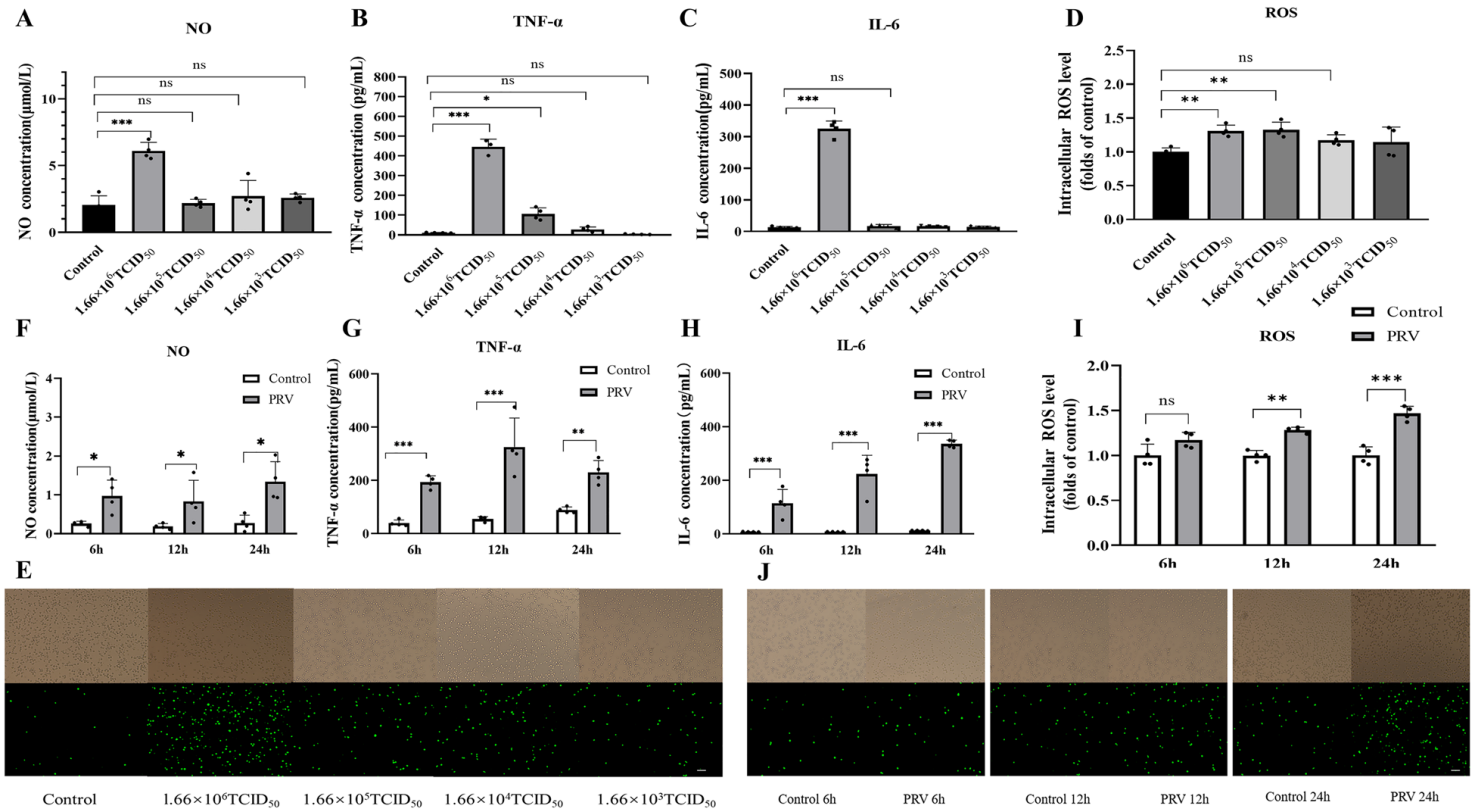
**Pseudorabies virus (PRV)-infected BV2 cells show increased production of inflammatory markers.** BV2 cells were infected with 1.66 × 10^6^ TCID_50_, 1.66 × 10^5^ TCID_50_, 1.66 × 10^4^ TCID_50_, and 1.66 × 10^3^ TCID_50_ PRV for 24 h. The levels of NO (**A**) in the supernatant were detected using the Griess method, the levels of TNF-α (**B**) and IL-6 (**C**) were detected using ELISA, and the levels of intracellular ROS (**D**, **E**) were detected by adding a fluorescent probe DCFH-DA. Furthermore, the levels of NO (**F**), TNF-α (**G**), IL-6 (H), and ROS (**I**, **J**) were measured at 6, 12, and 24 h after infection with 1.66 × 10^6^ TCID_50_ PRV_._ Green fluorescence indicates the presence of ROS in BV2 cells; scale bar = 200 μm. All experiments were performed in parallel. The results are presented as the mean ± standard deviation (SD) of four biological replicates (*n* = 4). Statistical significance was determined using one-way analysis of variance (ANOVA) followed by a least significant difference (LSD) post hoc test for multiple comparisons among the groups. *P < 0.05, **P < 0.01, ***P < 0.001, and NS, not significant.

After BV2 cells were infected with different titres of PRV for different times, the PRV-induced inflammatory response was assessed by measuring the levels of NO, TNF-α, IL-6, and ROS. We observed that NO, TNF-α, IL-6, and ROS levels were significantly increased in 1.66 × 10^6^ TCID_50_ PRV-infected BV2 cells at 24 h compared to those in the control group (Figures 1A–E). Furthermore, these levels were significantly higher in 1.66 × 10^6^ TCID_50_ PRV-infected BV2 cells at 6, 12, and 24 h than in the control group (Figures 1F–J). These results indicate that BV2 cells infected with 1.66 × 10^6^ TCID_50_ PRV produced inflammatory markers after 24 h of infection. Additionally, to exclude the effect of different cell numbers on the levels of inflammatory factors, the CCK-8 assay was used, and no changes in cell viability were observed in BV2 cells infected with 1.66 × 10^6^ TCID_50_ PRV after 24 h (Additional file 1).

### CUR suppresses and promotes the release of proinflammatory and anti-inflammatory cytokines, respectively, in PRV-infected BV2 cells

**Figure 2:**
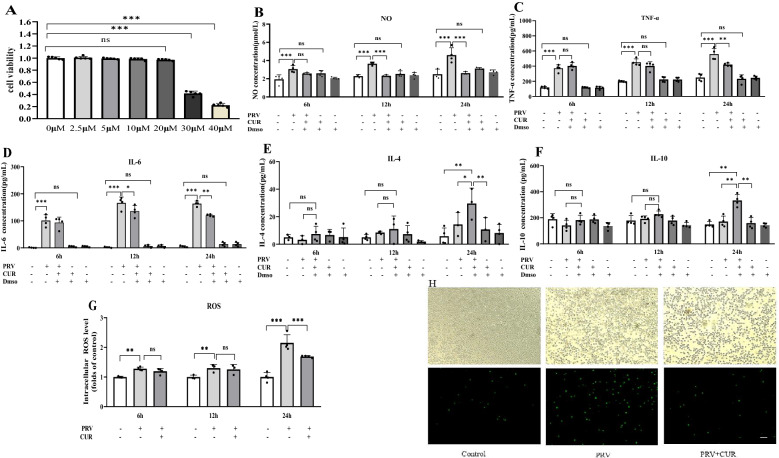
**Curcumin (CUR) suppresses proinflammatory cytokine release and promotes anti-inflammatory cytokine release in PRV-infected BV2 cells.** **A** BV2 cells were treated with different concentrations of CUR for 24 h, and cell viability was determined by the CCK-8 assay. BV2 cells were infected with/without 1.66 × 10^6^ TCID_50_ PRV for 24 h, followed by dimethyl sulfoxide (DMSO) or 20 μM CUR treatment for 6, 12, and 24 h. The levels of NO (**B**) in the supernatant were detected using the Griess method; the levels of TNF-α (**C**) and IL-6 (**D**), IL-4 (**E**), and IL-10 (**F**) were detected using ELISA; and the levels of intracellular ROS (**G**, **H**) were detected by adding the fluorescent probe DCFH-DA. Green fluorescence indicates the presence of ROS in BV2 cells; scale bar = 200 μm. All experiments were performed in parallel. The results are presented as the mean ± SD of four biological replicates (*n* = 4). Statistical significance was determined using one-way ANOVA followed by an LSD post hoc test for multiple comparisons among the groups. *P < 0.05, **P < 0.01, ***P < 0.001, and NS, not significant.

To determine whether CUR affected the viability of BV2 cells, the CCK-8 assay was performed after the cells were treated with 0–40 μM CUR for 24 h. The results revealed that CUR concentrations less than 20 μM did not induce any detectable cytotoxicity (Figure 2A). To ensure that CUR had the greatest effect, 20 μM CUR was used in the subsequent experiments.

Then, the optimal time point for the beneficial effects of CUR was determined. PRV treatment significantly increased NO, TNF-α, and IL-6 levels, whereas incubation with 20 μM CUR for 24 h significantly inhibited the release of NO (Figure 2B), TNF-α (Figure 2C), and IL-6 (Figure 2D) and significantly increased IL-4 (Figure 2E) and IL-10 (Figure 2F) levels. The addition of dimethyl sulfoxide (DMSO) and CUR alone within the dose range used in this study had no effect on the release of cytokines by BV2 cells compared to those in the control group. Therefore, control experiments with DMSO and CUR alone were not performed further. Subsequently, we measured the levels of ROS in BV2 cells and found that 20 μM CUR for 24 h inhibited the PRV-induced increase in ROS (Figures 2G, H).

The transformation of microglia from a ramified morphology to an amoeboid shape is associated with inflammation and neurotoxicity [35]. In this study, PRV-infected BV2 cells had an amoeboid shape with an enlarged cell body and had lost their extended processes. Pretreatment with CUR ameliorated the PRV-induced morphological changes in BV2 cells. Control and CUR only BV2 cells also showed a ramified morphology (Additional file 2). These results suggest that 20 μM CUR for 24 h inhibited PRV-induced proinflammatory cytokine production in BV2 cells.

### CUR promotes the polarization of PRV-infected BV2 cells from the M1 phenotype to the M2 phenotype and reverses PRV-induced mitochondrial dysfunction

**Figure 3:**
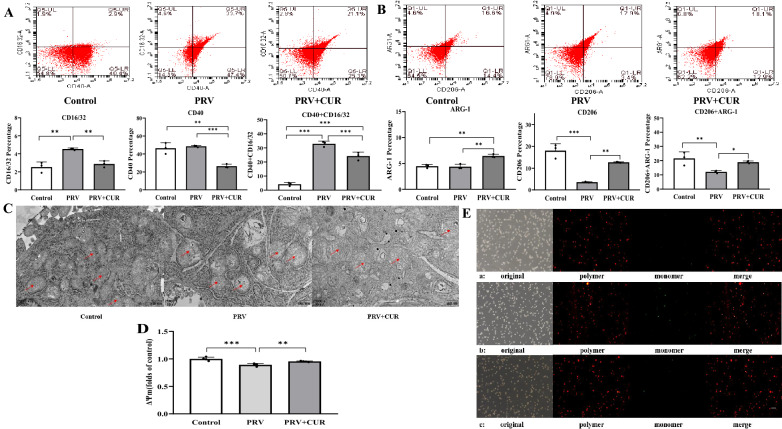
**CUR promotes polarization and reverses PRV-induced mitochondrial dysfunction in PRV-infected BV2 cells.** BV2 cells were infected with/without PRV for 24 h and then treated with/without 20 μM CUR for 24 h. **A** The expression of the M1 phenotype surface markers CD40 and CD16/32 and **B** the M2 phenotype surface markers CD206 and ARG-1 in BV2 cells was determined using flow cytometry (*n* = 3). **C** Mitochondrial structures in BV2 cells were observed using transmission electron microscopy (*n* = 3); scale bar = 500 nm. **D** Mitochondrial membrane potential (MMP) in BV2 cells. **E** MMP in BV2 cells was observed using fluorescence microscopy (*n* = 4); scale bar = 200 μm. All experiments were performed in parallel. The results are presented as the mean ± SD. Statistical significance was determined using one-way ANOVA followed by an LSD post hoc test for multiple comparisons among the groups. *P < 0.05, **P < 0.01, ***P < 0.001, and NS, not significant.

The expression levels of CD16/32, CD40, ARG-1, and CD206 were measured using flow cytometry to determine the effect of CUR on phenotypic switching in BV2 cells. The expression levels of M1 phenotypic markers such as CD16/32 and double-positivity for CD16/32 and CD40 were markedly upregulated by PRV infection compared with those in the control group (Figure 3A). Furthermore, CUR treatment greatly decreased the expression levels of CD16/32 and CD40 and double-positivity for CD16/32 and CD40 and markedly increased the expression of ARG-1 and CD206, which are markers of the M2 phenotype, and double-positivity for ARG-1 and CD206 in BV2 cells (Figure 3B). These results indicate that CUR induced the transformation of BV2 cells from the M1 to M2 phenotype.

An intact mitochondrial structure is a prerequisite for proper mitochondrial function. Mitochondrial dysfunction prevents the repolarization of inflammatory macrophages [36]. Therefore, to investigate whether CUR contributes to the restoration of mitochondrial function and improves the reprogramming of inflammatory macrophages to anti-inflammatory cells, TEM was used to observe mitochondrial structures. As shown in Figure 3C, most BV2 cells in the control group had clear and complete mitochondria and several long and uniform cristae that were neatly arranged and densely packed in the mitochondrial membrane. In contrast, the mitochondria of BV2 cells in the PRV-infected group were swollen and vacuolated, and cristae in the mitochondrial membrane were dissolved and broken. However, in the PRV + CUR treatment group, some mitochondria in BV2 cells displayed a clear and complete shape, and the dissolution and fragmentation of mitochondrial membrane cristae were weakened.

MMP is an important indicator of mitochondrial function. The JC-1 assay kit showed that the Δψm of the PRV group was significantly reduced compared with that of the control group. However, in the PRV + CUR group, the decrease in the Δψm was significantly reversed (Figures 3D, E). These results suggest that CUR reverses PRV-induced mitochondrial dysfunction.

### The secretions of CUR-treated BV2 cells protect neurons from PRV-induced apoptosis

**Figure 4:**
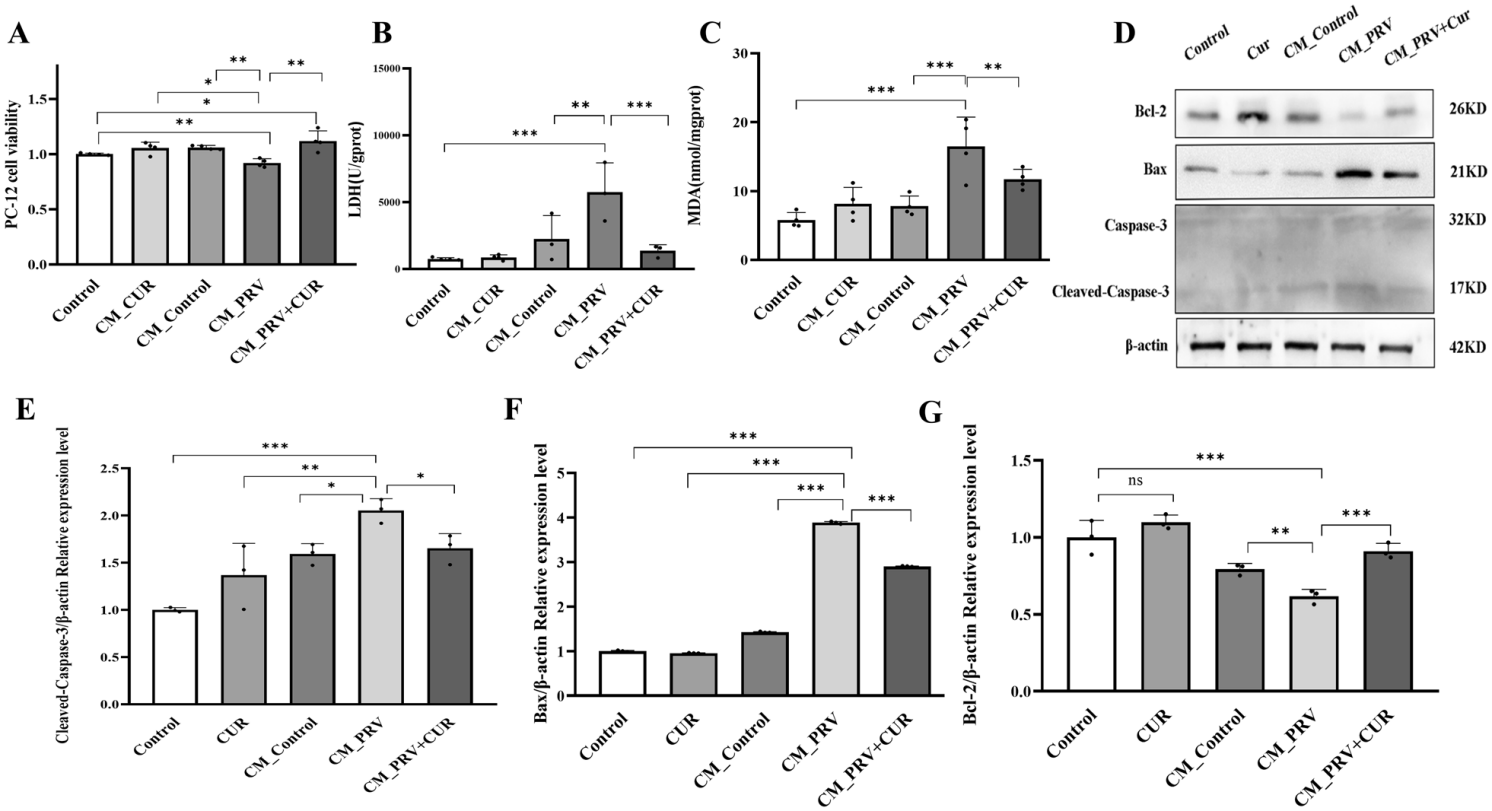
**CUR ameliorated neuronal apoptosis in PRV-infected BV2 cells.** PC-12 cells containing neuronal properties were treated with the supernatants of different phenotypes of BV2 cells for 24 h. **A** The viability of PC-12 cells (*n* = 4). **B** LDH activity in PC-12 cells (*n* = 3). **C** MDA levels in PC-12 cells (*n* = 3). **D** Representative Western blot analysis of apoptotic/antiapoptotic proteins; β-actin was used as the loading control (*n* = 3). **E** Relative protein levels of cleaved caspase-3. **F** Relative protein levels of Bax. **G** Relative protein levels of Bcl-2. All experiments were performed in parallel. The results are presented as the mean ± SD. Statistical significance was determined using one-way ANOVA followed by an LSD post hoc test for multiple comparisons among the groups. *P < 0.05, **P < 0.01, ***P < 0.001, and NS, not significant.

Activated microglia release neurotoxic agents, which correlate with the onset and progression of neurological diseases [37]. CUR inhibited the release of proinflammatory cytokines by PRV-infected BV2 cells. Therefore, we further determined whether the CM of CUR-treated cells could protect against PRV-induced neuronal toxicity in BV2 cells using CCK-8, LDH, and MDA assays. PC-12 cells were incubated with CM from BV2 cells for 24 h. Cell viability in the CM_Control and CM_CUR groups was similar to that in the control group. However, cell viability in the CM_PRV group was significantly decreased compared to that in the control group (Figures 4A–C). Notably, the viability of PC-12 cells in the CM_PRV + CUR group was significantly elevated compared to that in the CM_PRV group. Likewise, PC-12 cells in the control, CM_CUR, and CM_Control groups had fine morphology and smooth cell edges, while PC-12 cells exhibited axonal rupture and cell death in the CM_PRV group under a light microscope. However, axonal rupture in PC-12 cells was attenuated in the CM_PRV + CUR group (Additional file 3A). Annexin V-FITC/PI double staining showed that the numbers of apoptotic and dead PC-12 cells in the CM_Control and CM_CUR groups were similar to those in the control group. The CM-PRV group showed a significant increase in both of these cell types compared to the CM_Control and control groups. Furthermore, apoptosis and cell death in the CM_PRV + CUR group were lower than those in the CM_PRV group (Additional file 3B). Activated caspase-3 and Bax are key mediators of neurotoxin-induced neuronal apoptosis, whereas Bcl-2 is an antiapoptotic protein.

Then, we investigated whether the CM of CUR-treated cells could protect neurons from apoptosis induced by the secretions of PRV-infected BV2 cells by inhibiting caspase-3 activation and measured cleaved caspase-3 expression and activity using Western blotting. Consistent with the results obtained from the Annexin V-FITC/PI double staining assay, exposure of PC-12 cells to the CM of PRV-infected BV2 cells resulted in a substantial increase in cleaved caspase-3 and Bax protein levels and a substantial decrease in Bcl-2 protein levels. However, treatment with CM from PRV-infected BV2 cells treated with CUR significantly attenuated cleaved caspase-3 and Bax protein production and significantly increased Bcl-2 protein levels in PC-12 cells (Figures 4D–G). These results suggest that CUR attenuates neuronal apoptosis, which may be partially dependent on the regulation of microglial polarization and a reduction in inflammatory responses.

### Distinct mRNA signatures of BV2 cells with different phenotypes were identified using RNA-Seq analysis

**Figure 5:**
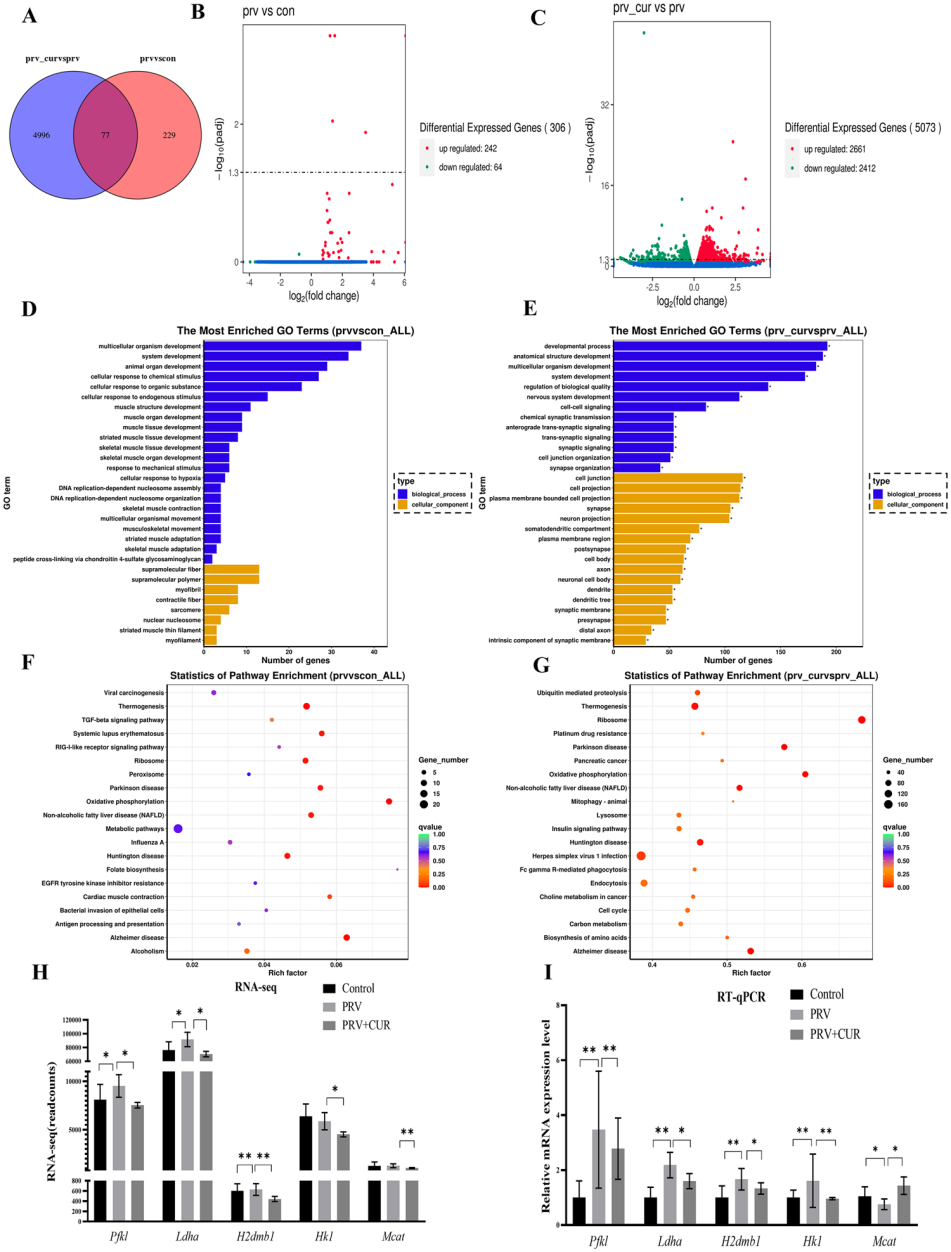
**Distinct mRNA signatures were identified during phenotypic transformation according to RNA-Seq analysis.** BV2 cells were infected with/without PRV for 24 h and then treated with/without 20 μM CUR for 24 h for RNA sequencing and analysis. Differentially expressed genes (DEGs) were detected by RNA-Seq analysis using DESeq2 (*n* = 3, *P* < 0.05, and fold change > 1). **A** Venn diagram showing DEGs. **B** DEG volcano plot of the PRV group vs. the control group. **C** DEG volcano plot of the PRV + CUR group vs. the PRV group. **D** GO enrichment analysis was performed on the DEGs in the PRV group vs. control group. **E** GO enrichment analysis was performed on the DEGs in the PRV + CUR group vs. PRV group. **F** KEGG enrichment analysis was performed on the DEGs in the PRV group vs. control group. **G** KEGG enrichment analysis was performed on the DEGs in the PRV + CUR group vs. PRV group. **D** KEGG enrichment analysis of DEGs. H) qRT‒PCR verification of differentially expressed genes identified in RNA-seq (*n* = 3). All experiments were performed in parallel. The results are presented as the mean ± SD. Statistical significance was determined using one-way ANOVA followed by an LSD post hoc test for multiple comparisons among the groups. *P < 0.05, **P < 0.01, ***P < 0.001, and NS, not significant.

Based on the abovementioned robust regulatory effects of CUR on cytokines in PRV-infected BV2 cells, we used RNA-seq to profile the transcriptomes of BV2 cells with different phenotypes (accession number: GSE201985). To obtain an unsupervised overview of the whole dataset, principal component analysis (PCA) was applied, and biological replicates were found to cluster together according to the PCA score plot (Additional file 4). This general pattern confirmed the reproducibility of the manipulations and the robustness of the data acquisition. Venn diagrams were used to show the numbers of DEGs in the PRV and control groups and in the PRV + CUR and PRV groups (Figure 5A). The analysis revealed 306 DEGs (*P* < 0.05, and fold change ≥ 1) in the PRV group compared with the control group, of which 242 genes were upregulated and 64 genes were downregulated (Figure 5B). Notably, a comparison of the PRV + CUR group with the PRV group identified 5,073 DEGs, of which 2661 genes were upregulated, and 2412 genes were downregulated (Figure 5C).

We performed GO and KEGG enrichment analyses on 306 DEGs in the PRV group relative to the control group and 5073 DEGs in the PRV + CUR group relative to the PRV group. As shown in Figures 5D and E, the results of GO analysis showed that both PRV vs. Control DEGs and PRV + CUR vs. PRV were significantly enriched in skeletal muscle-related biological processes, which may be related to the morphological plasticity of BV2 cells. According to the KEGG pathway enrichment analysis (Figure 5F, G), both PRV vs. Control and PRV + CUR vs. PRV DEGs were significantly enriched in metabolic pathways, oxidative phosphorylation, Alzheimer’s disease, and glycolysis/gluconeogenesis, suggesting that the phenotypic transformation of BV2 cells may be related to energy metabolism. To verify the reliability of the results, we performed qPCR analysis of some differentially expressed genes from the RNA-seq analysis (*Pfkl*, *Ldha, Hk1*, *Mcat*, and *H2dmb1*) that are glycolysis, FAS and phenotype-related genes. Our findings suggest that glycolysis and FAS in energy metabolism drive phenotypic shifts in PRV-infected BV2 cells (Figure 5H).

### CUR modulates phenotype-related cytokines in PRV-infected BV2 cells via AMPK-mediated energy metabolism-related pathways

**Figure 6:**
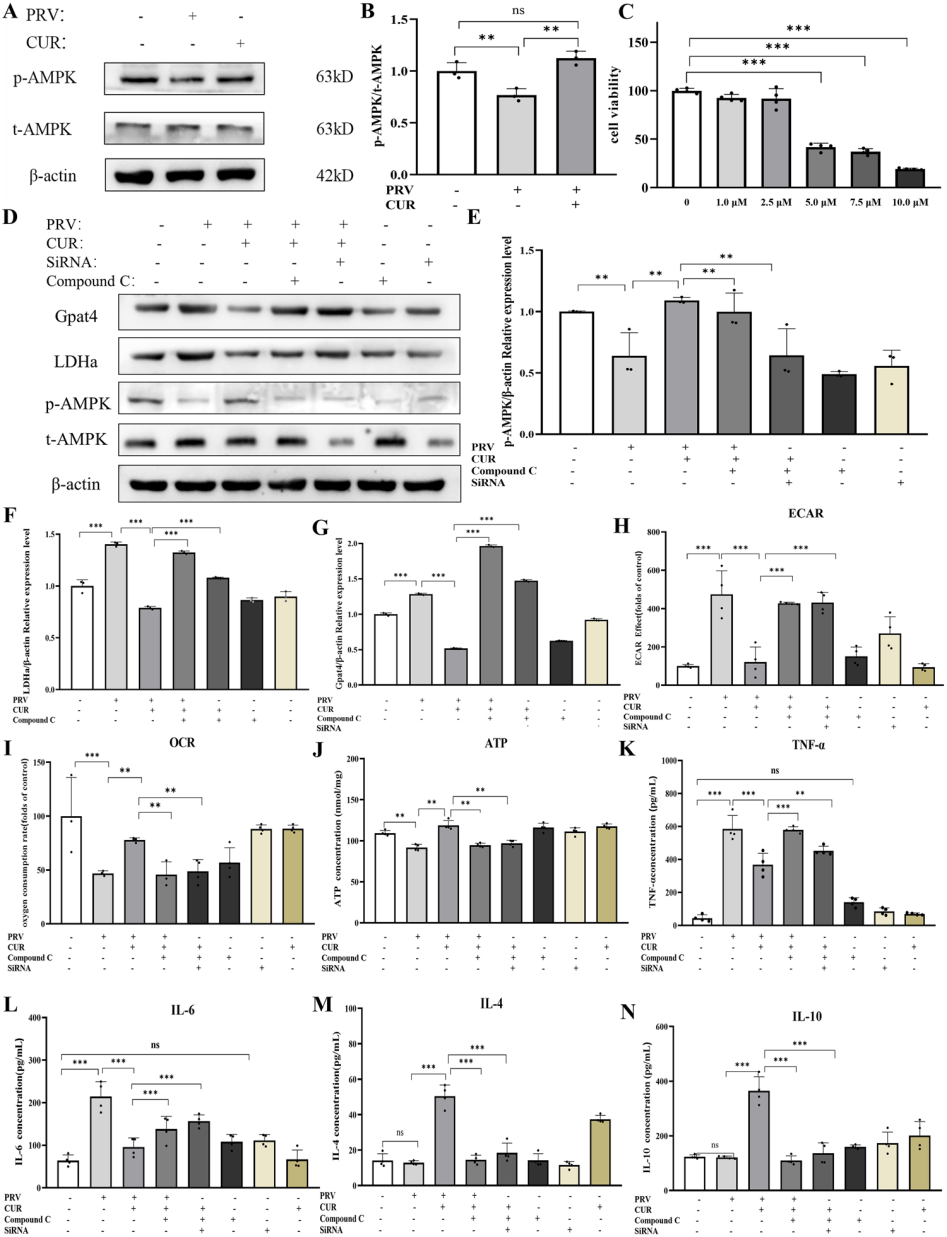
**CUR regulates energy metabolism through AMPK-dependent pathways in PRV-infected BV2 cells.** **A** BV2 cells were infected with/without PRV for 24 h and then treated with/without 20 μM CUR for 24 h. Western blot analysis of p-AMPK^Thr172^ protein levels (normalized to the t-AMPK protein). β-Actin was used as the loading control (*n* = 3). **B** Relative protein levels of p-AMPK^Thr172^. **C** BV2 cells were treated with different concentrations of Compound C for 24 h, and cell viability was determined by the CCK-8 assay to determine the toxicity range (*n* = 4). **D** BV2 cells were infected with/without PRV or were treated with PRV and an AMPK inhibitor (Compound C or siRNA) alone or in combination for 24 h, followed by treatment with/without 20 μM CUR for 24 h. Western blot analysis of p-AMPK (normalized to the t-AMPK protein), t-AMPK, Gpat4, and LDHa levels (Gpat4 and LDHa values were normalized to the β-actin protein); β-actin was used as the loading control (*n* = 3). **E** Relative p-AMPKThr172 protein levels. **F** Relative LDHa protein levels. **G** Relative Gpat4 protein levels. **H** The extracellular acidification rate of BV2 cells after the different treatments (values normalized to the control) (*n* = 4). **I** The OCR of BV2 cells after the different treatments (values normalized to the control) (*n* = 4). **J** BV2 cell ATP levels in the different treatment groups (*n* = 4). **K** The M1 phenotype-related inflammatory factor TNF-α in BV2 cells was detected using ELISA (*n* = 4). **L** The levels of the M1 phenotype-related inflammatory factor IL-6 in BV2 cells (*n* = 4). **M** The levels of the M2 phenotype-related anti-inflammatory factor IL-4 in BV2 cells (*n* = 4). **N** The levels of the M2 phenotype-related anti-inflammatory factor IL-10 in BV2 cells (*n* = 4). All experiments were performed in parallel. The results are presented as the mean ± SD. Statistical significance was determined using one-way ANOVA followed by an LSD post hoc test for multiple comparisons among the groups. *P < 0.05, **P < 0.01, ***P < 0.001, and NS, not significant.

During energy stress, activated AMPK directly phosphorylates key factors involved in multiple pathways to restore energy balance [38]. Accumulating evidence suggests that anti-inflammatory drugs can activate AMPK to alter energy metabolism in macrophages and exert anti-inflammatory effects [39, 40]. Therefore, AMPK phosphorylation was first analysed to determine whether the AMPK pathway was involved in the anti-inflammatory effect of CUR on PRV-infected BV2 cells. PRV infection significantly decreased the levels of phosphorylated AMPK. Conversely, CUR treatment abrogated the reduction in phosphorylated AMPK^T172^ levels (Figures 6A and B). To further assess whether AMPK regulates energy metabolism in PRV-infected microglia treated with CUR, BV2 cells were pretreated with/without the AMPK inhibitor Compound C or AMPK siRNA before the addition of CUR. As shown in Figure 6C, Compound C (≤ 2 μM) had no effect on the viability of BV2 cells. To ensure maximum suppression, 2 μM Compound C was used for further analyses. Fluorescence microscopy showed that the transfection efficiency of siRNA was good using (Additional file 5A), and the band with the best inhibitory effect was screened using Western blot analysis (Additional files 5B, C). Western blot analysis showed that CUR treatment abrogated the decrease in phosphorylated AMPK protein levels caused by PRV infection and inhibited the increase in LDHa (key enzyme in the glycolysis process) and Gpat4 (key enzyme in triacylglycerol synthesis) protein levels induced by PRV infection. Notably, Compound C or siRNA pretreatment reversed the effect of CUR on PRV-infected BV2 cells (Figure 6D–G). CUR enhanced the reductions in the OCR and ATP caused by PRV infection and attenuated the increase in the ECAR. Likewise, pretreatment with Compound C or siRNA reversed these effects of CUR (Figures 6H–J). Taken together, these results suggest that CUR regulates the activities of enzymes related to the energy metabolism pathways by activating AMPK, thereby increasing energy production and reducing the energy-consuming responses of PRV-infected BV2 cells.

To investigate the role of AMPK-related pathways in CUR-treated PRV-infected BV2 cells, the levels of microglial phenotype-related cytokines were measured using ELISA. As predicted, CUR inhibited the increases in TNF-α and IL-6 levels in the PRV-infected BV2 cells and promoted the production of IL-4 and IL-10. After pretreatment with Compound C or AMPK-α siRNA, the anti-inflammatory effects of CUR disappeared (Figures 6K–N). To further verify the role of CUR in transforming the phenotype of PRV-infected microglia by activating AMPK-energy metabolism-related pathways, we investigated the effect of Compound C and siRNA on CUR-treated primary cultured microglia. The purity of the microglia was identified by flow cytometry as 95.2% (Additional file 6A). Similar to previous findings, the effect of CUR disappeared after pretreatment with Compound C or siRNA (Additional file 6B).

These results show that CUR-treated PRV-infected microglia changed from the M1 phenotype to the M2 phenotype through activation of the AMPK-energy metabolism-related pathway.

### AMPK mediates the regulatory effect of CUR on NF-κB p65

**Figure 7:**
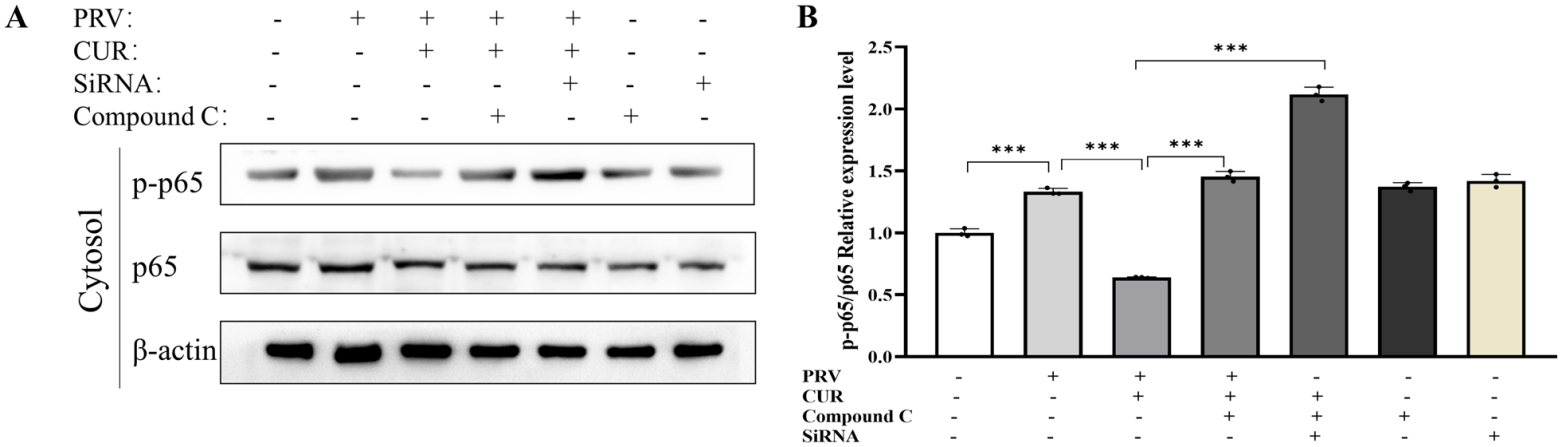
**NF-κB p65 may mediate the effects of CUR on the AMPK-energy response pathway.** BV2 cells were infected with/without PRV or treated with PRV and AMPK inhibitors (Compound C or siRNA) alone or in combination for 24 h, followed by treatment with/without 20 μM CUR for 24 h. **A** Western blot analysis of NF-κB p65 and NF-κB p-p65^Ser536^ levels; β-actin was used as the loading control (*n* = 3). **B** Relative p-p65^Ser536^ levels. All experiments were performed in parallel. The results are presented as the mean ± SD. Statistical significance was determined using one-way ANOVA followed by an LSD post hoc test for multiple comparisons among the groups. *P < 0.05, **P < 0.01, ***P < 0.001, and NS, not significant.

The proinflammatory NF-κB signalling pathway is a key regulator of immune processes, which affects changes in energy metabolism, such as OXPHOS, glycolysis, triglyceride levels, and lipogenesis [41]. Western blot analysis revealed that CUR treatment inhibited the increase in phosphorylated p65 levels in the cytoplasm in PRV-infected BV2 cells. However, pretreatment with Compound C or siRNA suppressed the phosphorylation of p65 (Figures 7A, B), indicating that CUR inhibits the NF-κB signalling pathway through AMPK.

### CUR ameliorates poor survival, CNS excitation, hyperthermia, and slow growth in PRV-infected rats

**Figure 8:**
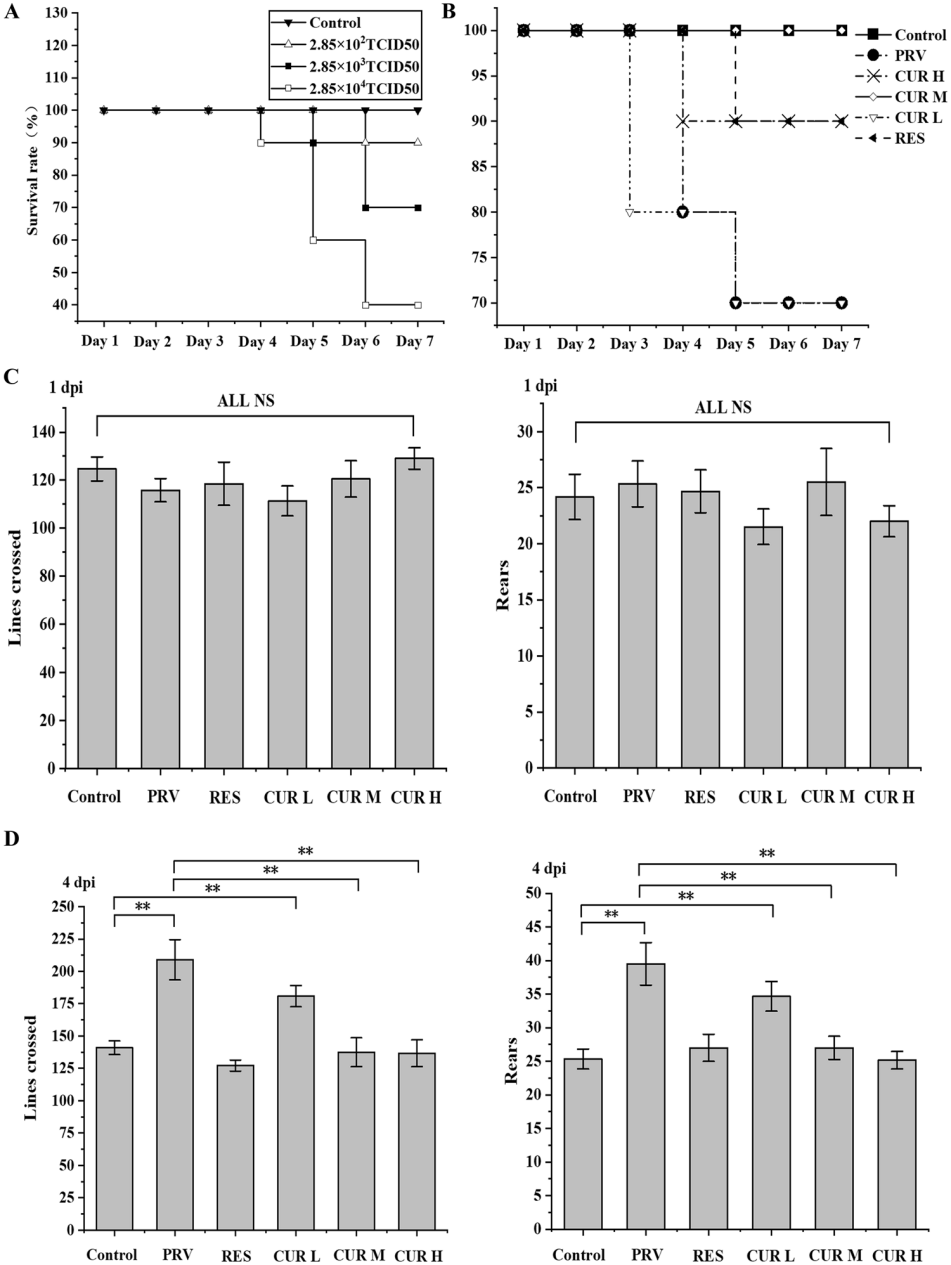
**CUR ameliorates central nervous system excitation, hyperthermia, slow growth, and poor survival in PRV-infected rats.** **A** After the rats were intraperitoneally injected with 2.85 × 10^2^ TCID_50_, 2.85 × 10^3^ TCID_50_ and 2.85 × 10^4^ TCID_50_ PRV or DMEM solution, the number of deaths in each group was recorded every day (*n* = 10). **B** The rats were intraperitoneally injected with low, medium, and high concentrations of CUR (25, 50, and 100 mg/kg BW) and resveratrol (RES 50 mg/kg BW) once per day for 14 days. On Day 8 (1 dpi), in addition to the control group, 0.1 mL of 2.85 × 10^3^ TCID_50_ PRV was injected, and the mortality was recorded daily. **C** The horizontal and vertical motor abilities of the rats were observed on the 8^th^ day (1 dpi). **D** The horizontal and vertical motor abilities of the rats were observed on Day 11 (4 dpi) (*n* = 3). All experiments were performed in parallel. The results are presented as the mean ± SD. Statistical significance was determined using one-way ANOVA followed by an LSD post hoc test for multiple comparisons among the groups. *P < 0.05, **P < 0.01, ***P < 0.001, and NS, not significant.

To determine the optimal titre of PRV infection, we injected rats with different titres of PRV and recorded the daily mortality. Rats infected with 2.85 × 10^2^ TCID_50_ PRV died at 6 dpi and had a survival rate of 90%. No deaths were observed after 7 dpi. Th Rats infected with 2.85 × 10^3^ TCID_50_ PRV began to die at 5 dpi, had a survival rate of 90%, and the survival rate was reduced to 70% at 6 dpi with no deaths recorded after 7 dpi. Rats infected with 2.85 × 10^4^ TCID_50_ PRV began to die at 4 dpi and had a survival rate of 90%, which decreased to 60% at 5 dpi. Mortality continued at 6 dpi with a reduced survival rate of 40%, and there were no further deaths at 7 dpi (Figure 8A). As the rats in the 2.85 × 10^3^ TCID_50_ PRV-infected group had a survival rate higher than 60% at 7 dpi and showed clinical symptoms, such as hyperactivity, increased body temperature, and weight loss, this titre was used as the infection dose for follow-up experiments.

To investigate the protective effect of CUR on PRV-infected rats, we intraperitoneally injected the rats with low, medium, and high concentrations of CUR. Since resveratrol has anti-inflammatory and anti-PRV properties [42], it was selected as a positive control in this study. The results indicated that there was no mortality in any group at 1 and 2 dpi. At 3 dpi, the survival rate in the CUR L group was 80%, while that in the other groups was 100%. At 4 dpi, the survival rates in the PRV and CUR L groups were 80%, that in the CUR H group was 90%, and those in the CUR M, RES, and control groups were 100%. At 5-7 dpi, the survival rates in the control, PRV, RES, CUR L, CUR M, and CUR H groups were 100%, 70%, 90%, 70%, 100%, and 90%, respectively (Figure 8B). An open-field test was used to explore the effect of CUR on PRV-induced central nervous system excitation in rats. At 1 dpi, there was no significant difference in the line crossing and rearing abilities of rats in each group. At 4 dpi, significant differences were observed in the line crossing and rearing abilities of the rats in the PRV-infected group and the low-dose CUR group (CUR L) compared with those in the control group; the line crossing and rearing abilities of the rats in the RES group, CUR medium-dose group (CUR M), and CUR high-dose group (CUR H) were significantly reduced compared to those in the PRV group (Figure 8C, D).

Then, we examined the effect of CUR treatment on body temperature and weight in PRV-infected rats. A prolonged body temperature increase occurred 3–5 days after PRV infection in rats, and low doses of CUR had no significant inhibitory effect on the PRV-induced increase in body temperature. However, treatment with resveratrol, medium-dose CUR, and high-dose CUR significantly inhibited the PRV-induced increase in body temperature (Table 1). PRV-infected rats displayed slow growth, and low-dose CUR did not significantly improve the growth inhibition caused by PRV, while treatment with resveratrol, medium-dose CUR, and high-dose CUR significantly improved PRV-induced slow growth (Table 2).

**Table 1:** **Daily variations in body temperature**

Day	Group					
	Control (℃)	PRV (℃)	RES (℃)	CUR L (℃)	CUR M (℃)	CUR H (℃)
Day 0	37.92 ± 0.06	37.88 ± 0.07	38.02 ± 0.06	37.98 ± 0.06	37.92 ± 0.07	37.98 ± 0.04
Day 1	38.00 ± 0.05	38.78 ± 0.22#	37.97 ± 0.05*	37.97 ± 0.11*	38.10 ± 0.13*	37.97 ± 0.09*
Day 2	38.07 ± 0.06	38.22 ± 0.13	38.00 ± 0.10	38.28 ± 0.18	37.90 ± 0.10*	37.85 ± 0.10
Day 3	37.87 ± 0.06	38.60 ± 0.09#	38.08 ± 0.12*	38.40 ± 0.10#	37.92 ± 0.14*	37.93 ± 0.09*
Day 4	38.05 ± 0.08	38.72 ± 0.14#	38.55 ± 0.11#	38.50 ± 0.06#	38.57 ± 0.11#	37.90 ± 0.08*
Day 5	37.85 ± 0.06	38.47 ± 0.08#	38.43 ± 0.06#	38.52 ± 0.11#	38.50 ± 0.06#	38.02 ± 0.15*
Day 6	38.00 ± 0.07	37.70 ± 0.14	38.57 ± 0.06#*	38.08 ± 0.17*	38.57 ± 0.08#*	38.62 ± 0.14#*
Day 7	37.85 ± 0.11	37.13 ± 0.13#	37.98 ± 0.08*	36.67 ± 0.47	38.03 ± 0.15*	38.00 ± 0.15*

Values represent the mean ± SD of 6 animals per group. #: the difference was significant compared with the control (*p* < 0.05), *: the difference was significant compared with the PRV group (*p* < 0.05).

**Table 2:** **The change in body weight gain in each group**

Time	Group					
	Control (g)	PRV(g)	RES(g)	CUR L(g)	CUR M(g)	CUR H(g)
Day 1	3.50 ± 0.55	3.12 ± 0.65	3.13 ± 0.25	3.67 ± 0.53	3.63 ± 0.70	3.50 ± 0.79
Day 2	7.60 ± 0.60	6.75 ± 0.63	7.30 ± 0.60	5.70 ± 0.45#	8.43 ± 0.73	8.05 ± 0.62
Day 3	12.83 ± 0.87	6.08 ± 0.32#	9.75 ± 0.85#*	5.43 ± 0.34#	11.50 ± 0.70*	11.48 ± 0.72*
Day 4	17.22 ± 1.05	3.27 ± 1.03#	12.13 ± 1.33#*	7.81 ± 0.70#*	14.87 ± 1.24*	14.12 ± 0.98*
Day 5	21.32 ± 1.17	3.55 ± 0.90#	14.65 ± 0.90#*	8.31 ± 0.95#*	16.83 ± 1.07#*	15.00 ± 1.34#*
Day 6	24.76 ± 1.03	3.88 ± 1.02#	16.3 ± 1.11#*	7.57 ± 1.08#*	19.13 ± 0.88#*	17.20 ± 1.15#*
Day 7	26.67 ± 1.12	4.77 ± 0.69#	17.95 ± 0.77#*	6.77 ± 0.82#	21.72 ± 0.67#*	18.50 ± 1.58#*

Values represent the mean ± SD of 6 animals per group. #: the difference was significant compared with the control (*p* < 0.05), *: the difference was significant compared with the PRV group (*p* < 0.05).

### CUR improves brain congestion, organ index changes, and vascular cuffing in PRV-infected rats

**Figure 9:**
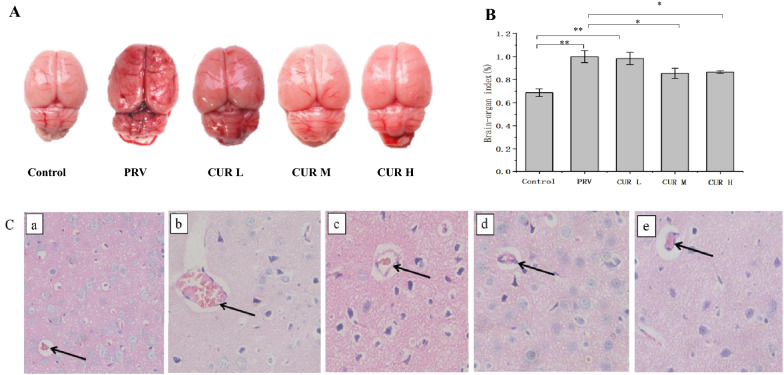
**CUR improves brain congestion, changes in organ indices, and vascular cuffing in PRV-infected rats.** Rats were intraperitoneally injected with low, medium, and high doses of CUR or 0.5% sodium carboxymethylcellulose solution (the solvent of CUR) for 7 consecutive days, and on the 8^th^ day, the rats were intraperitoneally injected with/without PRV. On the 14^th^ day (7 dpi), the rats in each group were sacrificed by decapitation, and the brain tissue was harvested for imaging to evaluate the organ index and prepare pathological sections. **A** The degree of blood congestion in the brain tissue in each group (*n* = 3). **B** Changes in the viscera index in rat brain tissue (*n* = 3). **C** Pathological sections of the cortex in each group, a: Control group, b: PRV group, c: CUR L group, d: CUR M group, e: CUR H group (*n* = 3), scale bar = 20 μm. All experiments were performed in parallel. The results are presented as the mean ± SD. Statistical significance was determined using one-way ANOVA followed by an LSD post hoc test for multiple comparisons among the groups. *P < 0.05, **P < 0.01, ***P < 0.001, and NS, not significant.

Rat brain tissues were used to observe the pathological changes caused by PRV infection. At 7 dpi, the brain tissues in the PRV and CUR L groups showed obvious swelling and congestion compared to those in the control group. However, brain tissue swelling and congestion in the CUR M and CUR H groups were obviously improved compared with those in the PRV group (Figure 9A). The CUR M and CUR H groups showed an increase in the brain tissue organ index compared to the PRV group (Figure 9B). Acute encephalitis is usually caused by pseudorabies virus [4]. Therefore, the vascular cuff phenomenon in PRV-infected rats was examined using HE staining. As expected, the PRV infection-induced vascular cuffing was ameliorated in the CUR L, CUR M, and CUR H groups (Figure 9C), indicating that CUR treatment ameliorated the cortical pathological changes and microglial activation induced by PRV infection.

## Discussion

PRV infection can cause viral encephalitis in animals and humans [4, 5], which is a devastating disease, and survivors often experience severe neurological complications. During viral encephalitis, dysregulated microglia produce cytokines that cause inflammatory damage to neurons, leading to neurocognitive impairment [43]. Therefore, the timely control of microglial activation may be an effective treatment strategy for viral encephalitis. First, we constructed an inflammatory model of PRV infection. Among the many proinflammatory cytokines, TNF-α, IL-6, and NO play major roles in the inflammatory response [44, 45]. Our study showed that PRV infection at 1.66 × 10^6^ TCID_50_ led to increased levels of TNF-α, IL-6, and NO in BV2 cells, indicating that an inflammatory response was induced. It has been reported that mitochondrial ROS are involved in metabolic changes associated with macrophage activation during inflammation, and M1 microglia generally show higher intracellular ROS levels than M0 and M2 cells [46, 47]. We found that PRV infection increased intracellular ROS levels in BV2 cells, suggesting that BV2 cells may switch from a resting M0 phenotype to an M1 phenotype. Correspondingly, we found that rat body temperature increased and body growth was reduced after PRV infection and microglial activation in vivo, which may be similar to the lipopolysaccharide-mediated induction of microglial inflammation [48]. Next, we investigated the optimal anti-inflammatory time point for CUR and whether it could transform the PRV-infected BV2 cell phenotype. We found that CUR treatment for 24 h reduced the secretion of the inflammatory factors TNF-α, IL-6, and NO, which was consistent with previous reports [20]. Eradicating the overproduction of ROS can mitigate proinflammatory M1 polarization and advance anti-inflammatory M2 polarization [47]. IL-4 and IL-10 can inhibit the production of proinflammatory cytokines, such as IL-8, IL-6, and TNF-α, and reduce the release of NO, thereby preventing neuronal damage induced by pathogenic microorganisms in vitro and in vivo [49]. In our study, CUR treatment attenuated the PRV-induced increase in intracellular ROS in BV2 cells and enhanced the secretion of IL-4 and IL-10, suggesting that CUR could convert PRV-infected BV2 cells from the M1 phenotype to the M2 phenotype. During viral encephalitis, the major group of MHC class II proteins, as well as the costimulatory molecules CD40, CD16, CD32, and CD86, are expressed on the surface of activated M1 microglia following viral infection [43], while M2 microglia typically express CD206 and ARG-1 [13]. Therefore, we hypothesized that CUR could convert the phenotype of PRV-infected BV2 cells, and our findings confirmed this hypothesis. Correspondingly, we found that CUR treatment significantly reversed the PRV infection-induced increase in body temperature, slow growth, and microglial activation in rats in vivo. The proinflammatory molecules TNF-α, IL-1β, CCL2, CCL5, and IL-6 have been reported to induce neuronal death by causing direct and indirect neurotoxicity [43, 50]. Therefore, to confirm that the changes in cytokines associated with the transformation of the microglial phenotype could alter the state of neuronal inflammatory damage, we first examined the effect of microglial supernatant on PC-12 cell activity. As a metabolite of lipid peroxidation, MDA often reflects the severity of ROS-induced cell damage. LDH is involved in glycolysis and is automatically released from the cell after cell damage. The degree of cell damage was assessed by measuring the amount of LDH released. Treatment of PC-12 cells with the supernatants of BV2 cells with different phenotypes for 24 h showed that the supernatant of M2 BV2 cell cells reversed the decrease in cell viability observed in response to M1 BV2 cell supernatant. We hypothesized that the decrease in cell viability may be caused by changes in the levels of apoptotic proteins. As expected, the supernatant of M2 BV2 cell cells reduced the number of apoptotic PC-12 cells, reduced the levels of the apoptotic proteins Bax and cleaved caspase-3, and increased the levels of Bcl-2 induced by the supernatant of M1 BV2 cells. Correspondingly, CUR treatment attenuated the PRV-induced excitation of the CNS, increased the tissue organ index and brain congestion, and improved the survival rates of the PRV-infected rats. The effects of CUR treatment on PRV-induced neuroinflammatory responses further validate the immunomodulatory role of CUR in the brain and provide new insights into the heterogeneous phenotype-specific microglial responses to this active small molecule.

Mitochondria are the main sites of energy metabolism and help cells carry out normal energy metabolism. Mitochondrial dysfunction prevents the repolarization of inflammatory macrophages [36, 51]. Therefore, the restoration of mitochondrial function can improve the reprogramming of inflammatory macrophages to anti-inflammatory cells. Our study showed that CUR treatment reversed PRV-induced mitochondrial swelling and vacuole formation, the dissolution of cristae in the mitochondrial membrane, and the increase in the number of fragmented mitochondria, indicating its effect on alleviating mitochondrial damage after infection. MMP is a key indicator of mitochondrial function. Lipopolysaccharide reduces MMP and mitochondrial damage in BV2 cells [51, 52]. Similarly, the present study showed that CUR treatment effectively ameliorated the PRV infection-induced reduction in MMP in BV2 cells. Thus, CUR ameliorated mitochondrial dysfunction in PRV-infected BV2 cells, which is a prerequisite for reprogramming inflammatory microglia into the anti-inflammatory M2 phenotype.

To further explore the mechanism by which CUR transforms PRV-infected BV2 cells at the transcriptomic level, we performed RNA-seq analysis of BV2 cells with M0, M1, and M2 phenotypes. Substantial gene expression changes occurred after PRV infection and CUR treatment. Compared with those in BV2 cells in the control group (M0 phenotype), glycolysis and proinflammatory-related genes (e.g., *Pfkl*, *Ldha*, and *H2dmb1*) were significantly upregulated in PRV-infected BV2 cells (M1 phenotype). However, CUR treatment significantly reduced the elevated levels of glycolysis and FAS-related genes (e.g., *Ldha*, *Hk-1*, *Gpat4*, and *Mcat*) induced by PRV infection (M2 phenotype). Furthermore, KEGG enrichment analysis of the gene subsets provided insight into the possible signalling pathways involved in the molecular mechanisms of phenotypic transformation. We found that the differentially expressed genes were mainly enriched in pathways related to energy metabolism (e.g., glycolysis, OXPHOS, and FAS). Therefore, we hypothesized that the change in the energy metabolism pathway in BV2 cells was closely related to phenotypic transitions. RT‒qPCR further verified the expression levels of genes associated with the AMPK pathway. AMPK is a highly conserved sensor of cellular energy status that can be activated by phosphorylation of its subunit at Thr172, which in turn affects energy metabolism [53] by regulating the expression of glycolysis pathway-related genes such as *Ldha*, *Hk1*, and *Pfkl* [54, 55] and fatty acid synthesis pathway-related genes such as *Gpat4* and *Mcat* [56–58]. It has been reported that p-AMPK levels are reduced during microglial inflammation [17]. Similarly, we found that the levels of p-AMPK were decreased after PRV infection, and CUR treatment reversed this decrease. When immune cells are activated, there is a shift in metabolism from OXPHOS to aerobic glycolysis, which is known as the Warburg effect [59]. The metabolism of M1 microglia mainly depends on aerobic glycolysis and the FAS pathway. Among these, two disruptions in the tricarboxylic acid (TCA) cycle lead to the accumulation of itaconic acid and succinic acid, which activate the transcription of glycolytic genes, thereby maintaining glycolytic metabolism in M1 microglia [12, 26]. Although ATP production efficiency is relatively low, ATP provides metabolic intermediates for FAS [26]. M2 microglia are more dependent on OXPHOS, and the TCA cycle is intact and provides substrates for complexes in the electron transport chain. This metabolic ATP production efficiency is relatively high, providing energy for the tissue repair and remodelling functions of M2 microglia [29]. LDH is a key enzyme in glycolysis, and its activity indirectly reflects glycolytic output during the metabolic reprogramming of BV2 cells [12]. The OCR reflects cellular OXPHOS levels; GPAT4 is involved in the synthesis of triacylglycerol [60]. Our study showed that CUR treatment reversed the PRV infection-induced increases in the protein levels of LDHa, GPAT4, and ECAR and increased the OCR and ATP levels. However, after AMPK activity was knocked down by an AMPK inhibitor and siRNA, the beneficial effect of CUR was not observed, indicating that the effect of CUR was mediated by AMPK. The NF-κB pathway is a key regulator of immune processes and has long been regarded as a typical proinflammatory signalling pathway. In recent years, this pathway has been reported to affect changes in energy metabolism related to inflammation and immune responses (e.g., OXPHOS and glycolysis) [41]. NF-κB binds to the inhibitor molecule IκB to form a p50-p65-IκB trimer under steady state conditions. In response to inflammatory mediators, the NF-κB subunit p65 is phosphorylated at Ser536 and migrates into the nucleus, upregulating the expression of various inflammation-related genes [61]. We therefore examined whether AMPK regulates energy metabolism through NF-κB p-65. The results showed that CUR reversed the increase in p-p65 levels caused by PRV infection. After AMPK activity was knocked down, the effects of CUR disappeared, suggesting that NF-κB may regulate energy metabolism via AMPK. Finally, we examined inflammatory factors associated with different phenotypes in BV2 cells, validated them in primary microglia, and concluded that CUR was required for phenotypic transition by repairing mitochondrial dysfunction, microglial phenotypic transition was driven by the AMPK energy metabolism pathway, and NF-κB may mediate this process.

In conclusion, to our knowledge, this is the first report on CUR-mediated resistance to PRV-induced encephalitis through the modulation of phenotypic transitions mediated by the AMPK/NF-κB-energy metabolism signalling pathway in microglia. Our study provides insight into the beneficial effects of CUR treatment on PRV-induced neuroinflammation.

## Supplementary Information

**Additional file 1. The effect of 1.66 × 10**^**6**^** TCID**_**50**_** PRV infection for 24 h on the viability of BV2 cells.** The cells were infected with 1.66 × 106 TCID_50_ PRV for 24 h, and the changes in the survival rates were measured. All experiments were performed in parallel. The results are expressed as the mean ± standard deviation (SD) of four biological replicates (n = 4). Statistical significance was determined using a two-tailed independent t test to compare the two groups. *P < 0.05, **P < 0.01, ***P < 0.001, and NS, not significant.

**Additional file 2. Effect of CUR on the morphology of PRV-infected BV2 cells.** (**A**) Untreated BV2 cells. (**B**) BV2 cells were infected with 1.66 × 10^6^ TCID_50_ PRV for 24 h, and the cell maintenance medium was replaced. (**C**) BV2 cells were infected with 1.66 × 10^6^ TCID_50_ PRV for 24 h and then treated with 20 μM curcumin (CUR) for 24 h. Morphological changes in BV2 cells were observed under a light microscope (scale bar = 200 μm). All experiments were performed in parallel.

**Additional file 3. Effects of the supernatants of BV2 cells with different phenotypes on PC-12 cell morphology and apoptosis.** The supernatants of BV2 cells with different phenotypes were added to PC-12 cells and incubated for 24 h. (**A**) Morphological changes in PC-12 cells were observed using a light microscope (scale bar = 200 μm). (**B**) Apoptosis in PC-12 cells detected using the Annexin V-FITC/PI kit. Red fluorescence represents late apoptotic and dying cells, while green fluorescence represents early apoptotic cells; scale bar = 200 μm. All experiments were performed in parallel.

**Additional file 4. Principal component analysis (PCA).** (**A**) Correlation check of the RNA-seq data using Pearson’s Correlation Coefficient. R^2^ ≥ 0.8 represents the repeatability of the experiment and the reliability of the evaluation results.

**Additional file 5. Screening the optimal interference effect of different siRNAs.** (**A**) The effects of siRNA transfection were observed under a fluorescence microscope. PC-siRNA group, positive control group; NC-FAM-siRNA group, fluorescence-labelled negative control group; FAM-siRNA 1 group, fluorescently labelled small interfering RNA first band group; FAM-siRNA 2 group, fluorescently labelled small interfering RNA second band group; FAM-siRNA 3 group, fluorescently labelled small interfering RNA third band group. (**B**) Western blot analysis of t-AMPK protein levels; β-actin was used as a loading control (*n* = 3). (**C**) Relative protein levels of t-AMPK. All experiments were performed in parallel. The results are presented as the mean ± SD. Statistical significance was determined using one-way analysis of variance (ANOVA) followed by a least significant difference (LSD) post hoc test for multiple comparisons among the groups. *P < 0.05, **P < 0.01, ***P < 0.001, and NS, not significant.

**Additional file 6. Purity of primary microglia and the effects of curcumin on the secretion of pro- and anti-inflammatory cytokines by PRV-infected microglia.** (**A**) Microglial purity was identified by flow cytometry; CD11b indicated resting microglia, and MHC Class II indicated activated cells (*n* = 3). (**B**) M1 phenotype-related inflammatory factors (TNF-α) in primary microglial cells were examined using ELISA (*n* = 4). (**C**) Levels of M1 phenotype-related inflammatory factors (IL-6) in primary microglial cells (*n* = 4). (**D**) Levels of M2 phenotype-related anti-inflammatory factors (IL-4) in primary microglial cells (*n* = 4). (E) Levels of M2 phenotype-related anti-inflammatory factors (IL-10) in primary microglial cells (*n* = 4). (*n* = 4). All experiments were performed in parallel. The results are presented as the mean ± SD. Statistical significance was determined using one-way ANOVA followed by an LSD post hoc test for multiple comparisons among the groups. *P < 0.05, **P < 0.01, ***P < 0.001, and NS, not significant.

## Data Availability

The data analysed during the current study are available from the corresponding author upon reasonable request.
