# Supplementary figures and images for: Curcumin-dependent phenotypic transformation of microglia mediates resistance to pseudorabies-induced encephalitis
Source: Vet Res. 2023 Mar 14;54:25. doi: 10.1186/s13567-023-01149-x (PMC10015794; doi:10.1186/s13567-023-01149-x)

**
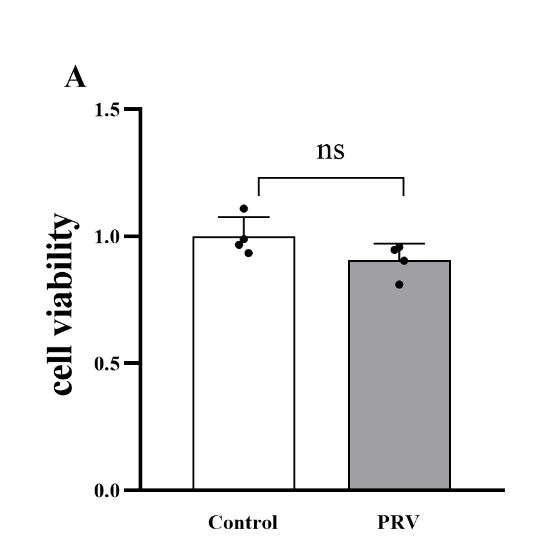
**

Supplement: Supplementary file 1 — Additional file 1. The effect of 1.66 × 10^6^ TCID50 PRV infection for 24 h on the viability of BV2 cells. The cells were infected with 1.66 × 10^6^ TCID50 PRV for 24 h, and the changes in the survival rates were measured. All experiments were performed in parallel. The results are expressed as the mean ± standard deviation (SD) of four biological replicates (n = 4). Statistical significance was determined using a two-tailed independent t test to compare the two groups. *P < 0.05, **P < 0.01, ***P < 0.001, and NS, not significant. [file 13567_2023_1149_MOESM1_ESM.docx]

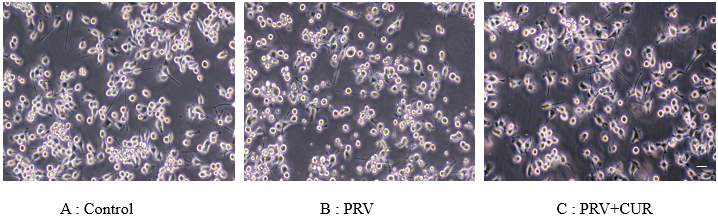

Supplement: Supplementary file 2 — Additional file 2. Effect of CUR on the morphology of PRV-infected BV2 cells. (A) Untreated BV2 cells. (B) BV2 cells were infected with 1.66 × 10^6^ TCID50 PRV for 24 h, and the cell maintenance medium was replaced. (C) BV2 cells were infected with 1.66 × 10^6^ TCID50 PRV for 24 h and then treated with 20 μM curcumin (CUR) for 24 h. Morphological changes in BV2 cells were observed under a light microscope (scale bar = 200 μm). All experiments were performed in parallel. [file 13567_2023_1149_MOESM2_ESM.docx]

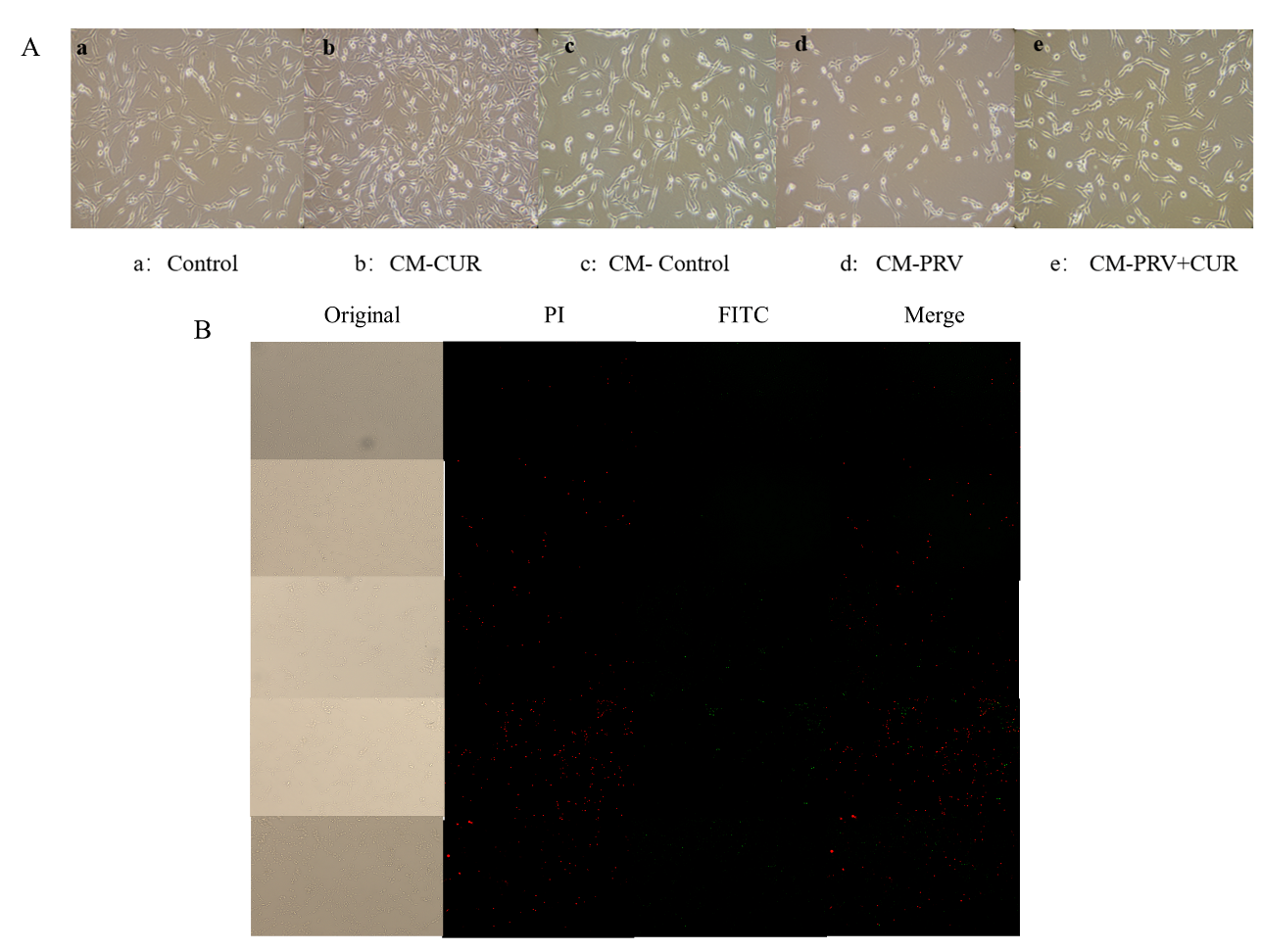

Supplement: Supplementary file 3 — Additional file 3. Effects of the supernatants of BV2 cells with different phenotypes on PC-12 cell morphology and apoptosis. The supernatants of BV2 cells with different phenotypes were added to PC-12 cells and incubated for 24 h. (A) Morphological changes in PC-12 cells were observed using a light microscope (scale bar = 200 μm). (B) Apoptosis in PC-12 cells detected using the Annexin V-FITC/PI kit. Red fluorescence represents late apoptotic and dying cells, while green fluorescence represents early apoptotic cells; scale bar = 200 μm. All experiments were performed in parallel. [file 13567_2023_1149_MOESM3_ESM.docx]

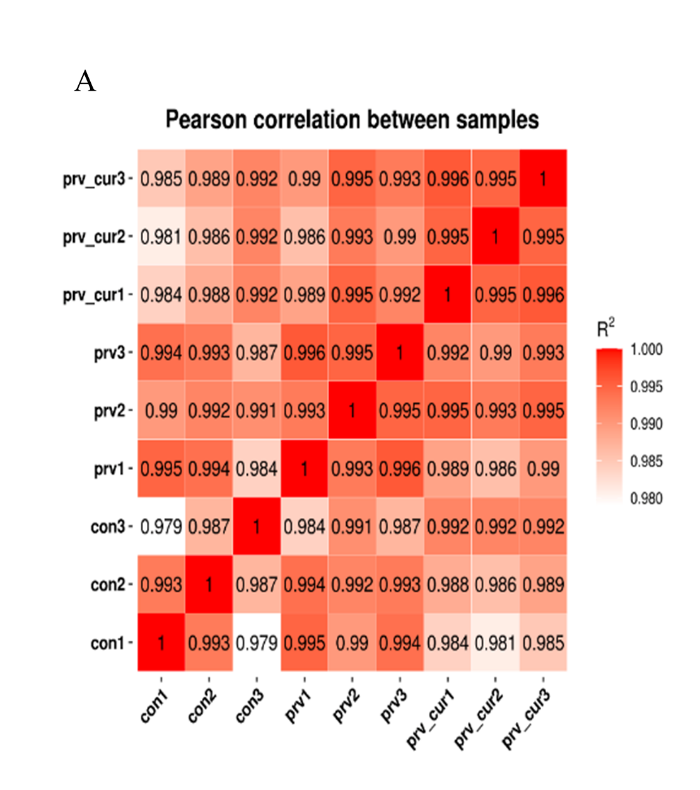

Supplement: Supplementary file 4 — Additional file 4. Principal component analysis (PCA). (A) Correlation check of the RNA-seq data using Pearson’s Correlation Coefficient. R2 ≥ 0.8 represents the repeatability of the experiment and the reliability of the evaluation results. [file 13567_2023_1149_MOESM4_ESM.docx]

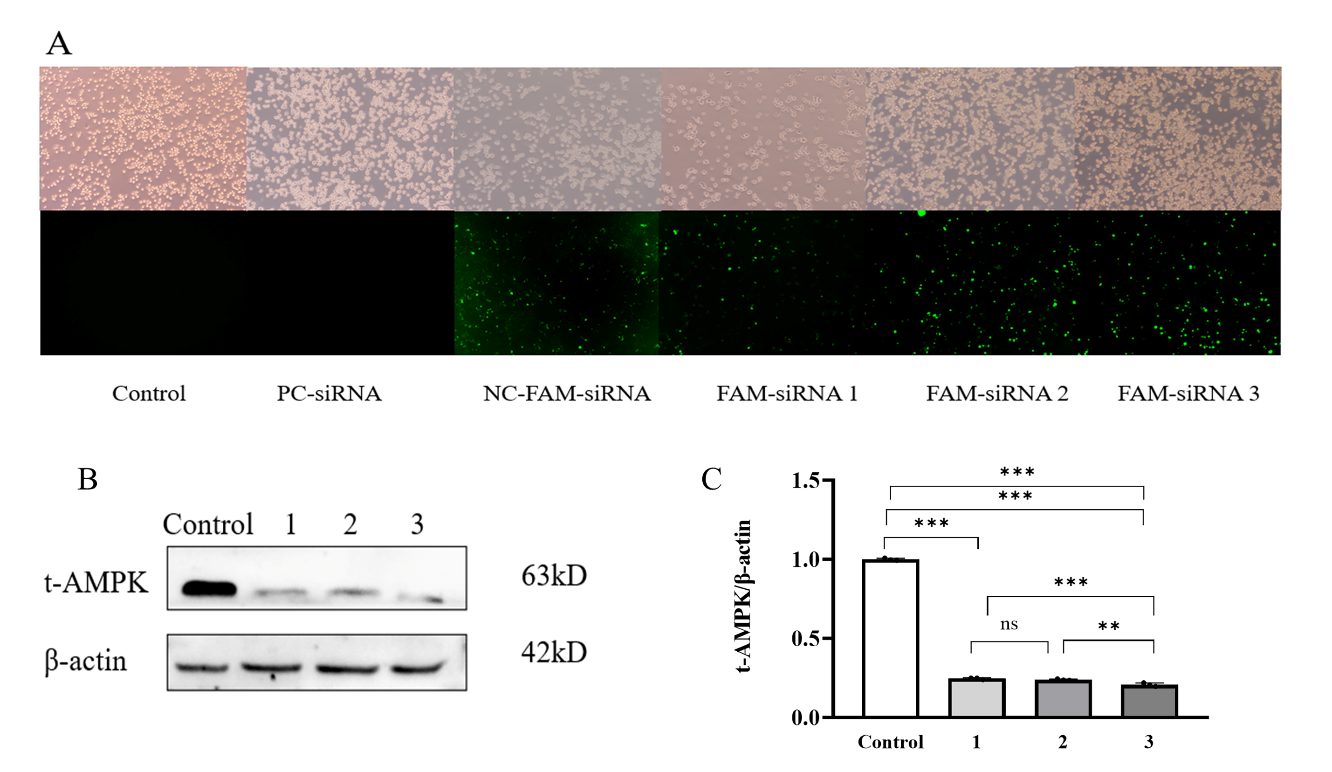

Supplement: Supplementary file 5 — Additional file 5. Screening the optimal interference effect of different siRNAs. (A) The effects of siRNA transfection were observed under a fluorescence microscope. PC-siRNA group, positive control group; NC-FAM-siRNA group, fluorescence-labelled negative control group; FAM-siRNA 1 group, fluorescently labelled small interfering RNA first band group; FAM-siRNA 2 group, fluorescently labelled small interfering RNA second band group; FAM-siRNA 3 group, fluorescently labelled small interfering RNA third band group. (B) Western blot analysis of t-AMPK protein levels; β-actin was used as a loading control (n = 3). (C) Relative protein levels of t-AMPK. All experiments were performed in parallel. The results are presented as the mean ± SD. Statistical significance was determined using one-way analysis of variance (ANOVA) followed by a least significant difference (LSD) post hoc test for multiple comparisons among the groups. *P < 0.05, **P < 0.01, ***P < 0.001, and NS, not significant. [file 13567_2023_1149_MOESM5_ESM.docx]

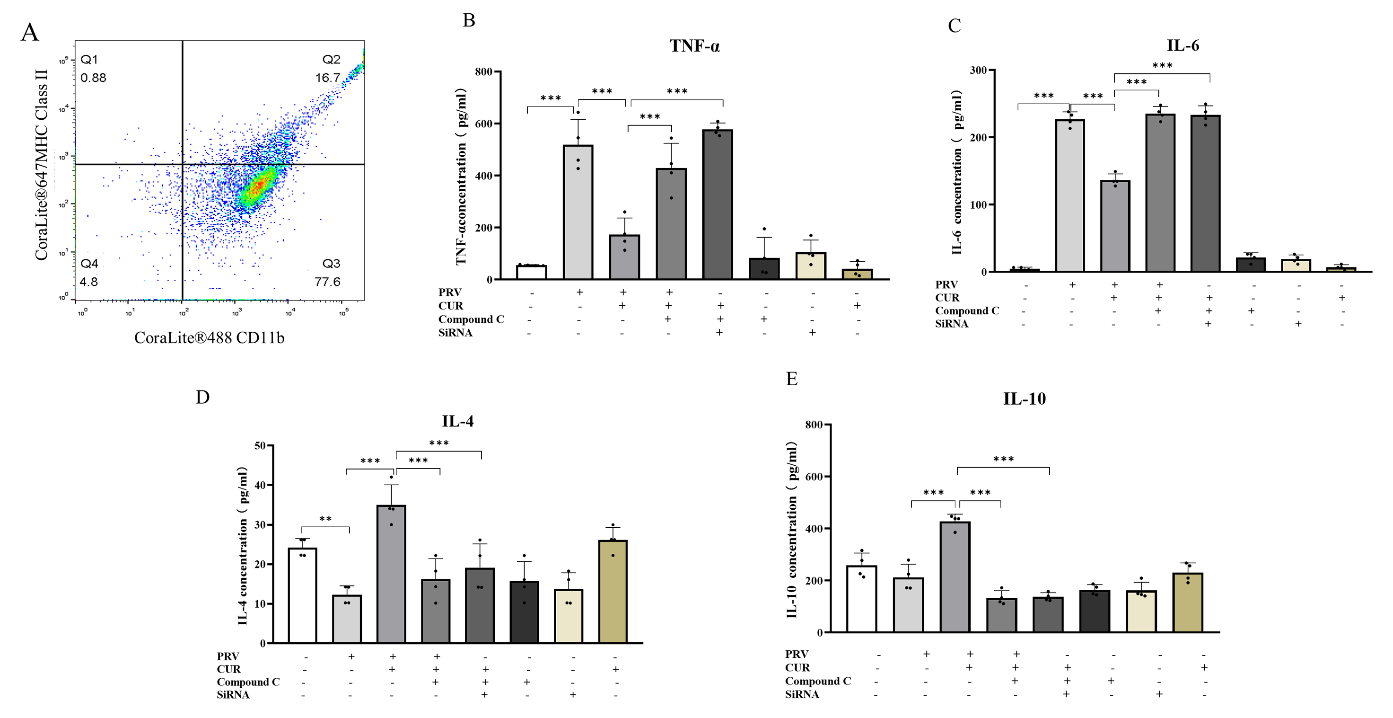

Supplement: Supplementary file 6 — Additional file 6. Purity of primary microglia and the effects of curcumin on the secretion of pro- and anti-inflammatory cytokines by PRV-infected microglia. (A) Microglial purity was identified by flow cytometry; CD11b indicated resting microglia, and MHC Class II indicated activated cells (n = 3). (B) M1 phenotype-related inflammatory factors (TNF-α) in primary microglial cells were examined using ELISA (n = 4). (C) Levels of M1 phenotype-related inflammatory factors (IL-6) in primary microglial cells (n = 4). (D) Levels of M2 phenotype-related anti-inflammatory factors (IL-4) in primary microglial cells (n = 4). (E) Levels of M2 phenotype-related anti-inflammatory factors (IL-10) in primary microglial cells (n = 4). (n = 4). All experiments were performed in parallel. The results are presented as the mean ± SD. Statistical significance was determined using one-way ANOVA followed by an LSD post hoc test for multiple comparisons among the groups. *P < 0.05, **P < 0.01, ***P < 0.001, and NS, not significant. [file 13567_2023_1149_MOESM6_ESM.docx]
